# UHPLC-(ESI)-HRMS and NMR-Based Metabolomics Approach to Access the Seasonality of *Byrsonima intermedia* and *Serjania marginata* From Brazilian Cerrado Flora Diversity

**DOI:** 10.3389/fchem.2021.710025

**Published:** 2021-07-06

**Authors:** Ana C. Zanatta, Wagner Vilegas, RuAngelie Edrada-Ebel

**Affiliations:** ^1^ Laboratory of Phytochemistry, Institute of Chemistry, Department of Biochemistry and Organic Chemistry, São Paulo State University (UNESP), Araraquara, Brazil; ^2^ Laboratory of Bioprospecting of Natural Products, Institute of Biosciences, São Paulo State University (UNESP), São Vicente, Brazil; ^3^ Strathclyde Institute of Pharmacy and Biomedical Sciences, University of Strathclyde, Glasgow, United Kingdom

**Keywords:** metabolomic approach, specialized metabolites, seasonality, environmental factors, phenolic compounds, saponins, triterpenes

## Abstract

Seasonality is one of the major environmental factors that exert influence over the synthesis and accumulation of secondary metabolites in medicinal plants. The application of the metabolomics approach for quality control of plant extracts is essentially important because it helps one to establish a standard metabolite profile and to analyze factors that affect the effectiveness of the medicinal plants. The Brazilian Cerrado flora is characterized by a rich diversity of native plant species, and a number of these plant species have been found to have suitable medicinal properties. Some of these plant species include *Byrsonima intermedia* and *Serjania marginata*. To better understand the chemical composition of these plant species, we conducted a study using the state-of-the-art techniques including the HPLC system coupled to an Exactive-Orbitrap high resolution mass spectrometer with electrospray ionization interface UHPLC-(ESI)-HRMS and by NMR being performed 2D *J*-resolved and proton NMR spectroscopy. For the analysis, samples were harvested bimonthly during two consecutive years. UHPLC-(ESI)-HRMS data were preprocessed and the output data uploaded into an in-house Excel macro for peak dereplication. MS and NMR data were concatenated using the data fusion method and submitted to multivariate statistical analysis. The dereplication of LC-HRMS data helped in the annotation of the major compounds present in the extracts of the three plant species investigated allowing the annotation of 68 compounds in the extracts of *B. intermedia* (cinnamic acids, phenolic acids derived from galloyl quinic and shikimic acid, proanthocyanidins, glycosylated flavonoids, triterpenes and other phenols) and 81 compounds in the extracts of *S. marginata* (phenolic acids, saponins, proanthocyanidins, glycosylated flavonoids among other compounds). For a better assessment of the great number of responses, the significance of the chemical variables for the differentiation and correlation of the seasons was determined using the variable importance on projection (VIP) technique and through the application of the false discovery rate (FDR) estimation. The statistical data obtained showed that seasonal factors played an important role on the production of metabolites in each plant species. Temperature conditions, drought and solar radiation were found to be the main factors that affected the variability of phenolic compounds in each species.

## Introduction

Natural products derived from the secondary metabolism of plants have played an essentially important role in the treatment of diseases and illnesses throughout the life history of humans. The search for relief and cure of diseases through the ingestion of herbs and leaves may have been one of the earliest ways in which natural products were used (Cragg and Newman, 2013; Katz and Baltz, 2016). The diversity of molecular structures and nature's ability to provide molecules of structural complexity hardly imagined or elaborated by synthesis is the result of many different biosynthetic pathways that are involved in the production of a plant's secondary metabolites, and what also leads these metabolites to exhibit a wide range of biological activities (Harvey et al., 2015; Bernardini et al., 2018).

The production of specialized metabolites by plants is characterized by the specificity of the plant species and is regarded an essentially relevant adaptive response in terms of coping with environmental and/or external stimuli to which the plants are exposed (Ghorbanpour and Varma, 2017). In addition, these metabolites play a key role in regulating plants growth, development, and defense, as well as in mediating plant-environment interactions, including protection against several stressful and challenging environmental change conditions (Rai et al., 2017; Isah, 2019; Pandey and Senthil-Kumar, 2019; Erb and Kliebenstein, 2020).

In recent decades, there has been an increasingly growing scientific interest in gaining a comprehensive understanding regarding the changes in secondary metabolism in plants, and a considerable number of studies have been conducted aiming at investigating the effects of changes in environmental factors (e.g., temperature, climate change, precipitation, drought, salinity, UV radiation, light and humidity) and seasonality on the biosynthesis and accumulation of specialized metabolites in medicinal plants (Xiao et al., 2008; Sampaio et al., 2016; Berini et al., 2018; Yang et al., 2018). For purposes of illustration, Sehlakgwe et al. (2020) demonstrated the effect of seasonal variation on the chemical profile of plants and the differences in activity observed for the plant material harvested in different seasons; the authors showed that winter was the best harvest period for *Leucosidea sericea* leaves. In another study, Botha et al. (2018) investigated the variations in the accumulation of three secondary metabolites present in different parts of the *Euclea undulata* Thunb. var. *myrtina* plant and the correlation with the amount of rainfall during the dry and rainy seasons, as well as changes in temperature.

As aforementioned, due to several factors, the content of specialized metabolites can vary considerably in plants species; and since many of these metabolites are active principles, this variation can eventually change the therapeutic response of the medicinal plant when applied for particular pharmacological purposes. In view of that, studying the effects of factors that may determine or modify the yield of these compounds in plants, such as growing and/or harvesting conditions, season and time of day, is extremely important if one aims to obtain good quality herbal products with desirable concentrations of active ingredients (Kunle et al., 2012; Liebelt et al., 2019).

Conducting a rapid, high-quality analysis of the chemical constituents of a complex plant matrix is indispensable in the sense that it allows one to characterize the rationally active markers in the matrix and facilitates one’s understanding of the relationship between the chemical composition and the possible efficacy, toxicity and therapeutic target of plant-based medicine (Wolfender et al., 2019).

Remarkably, a major obstacle to establishing a reliable chemical quality control framework of a plant extract is that plants have an extremely complex composition with a huge number of compounds, of which there is still very limited knowledge. The chemical diversity of compounds present in medicinal plants is directly associated with the high variability of the intrinsic physicochemical properties of natural products, and this makes the separation, detection and identification of the natural substrates of the plants technically challenging (Dunn et al., 2011; Wolfender et al., 2015).

Significant advances in analytical techniques along with new bioinformatics tools and multivariate statistical analysis have contributed meaningfully toward the advancement of chemical studies of complex samples. Metabolomics-based approaches which employ state-of-the-art techniques, such as high resolution mass spectrometry (HRMS) and one and two-dimensional NMR spectroscopy, is a fast and efficient way of maximizing the results of metabolic fingerprinting analysis of a large number of data sets (Kellogg et al., 2017; Han et al., 2018; Sut et al., 2019; Houriet et al., 2020). Furthermore, unlike their individual application, the combined application of MS and NMR (MS-NMR) provides more reliable identification results and unbiased assessment of quality control analyses of medicinal plants in addition to helping predict the bioactivity of the plants (Sampaio et al., 2016; Sut et al., 2019; Sehlakgwe et al., 2020).

The Brazilian Cerrado flora is widely known to be constituted by a rich diversity of plant species, and a number of these plant species have been found to possess suitable medicinal properties (Lahsen et al., 2016; Cortelo et al., 2021). Some of these plant species include *Byrsonima intermedia* and *Serjania marginata*, which present similar ethnopharmacological characteristics and have been found to be promising for future application in phytotherapy aimed at the treatment of inflammatory diseases. The hydroethanolic extracts obtained from the leaves of each of these species demonstrated potential gastroprotective (Arruda et al., 2009; Périco et al., 2015; dos Santos et al., 2019), anti-inflammatory (Moreira et al., 2011; Salinas-Sánchez et al., 2017), antioxidant (Heredia-Vieira et al., 2015; Guilhon-Simplicio et al., 2017), analgesic (Di Stasi et al., 1988; Verdam et al., 2017) activities and absence of toxicity in the acute models evaluated (Santos et al., 2012; Périco et al., 2015; dos Santos et al., 2019).

The *B. intermedia* A. Juss species is popularly known as “murici-do-cerrado”, “cajuzinho do cerrado” or "murici-mirim" - this name, which means a small tree in Tupi Guarani language, was given to the species by the indigenous people. The *B. intermedia* A. Juss species is native to the Brazilian Cerrado (the Brazilian tropical savanna region), and as the name implies, the plant species has a bushy-like characteristics and has been found to reach a maximum height of 1.60 m. The local population of this savanna uses the bark and leaves of *B. intermedia* as infusions; these infusions have been found to possess antiseptic, antimicrobial, anti-haemorrhagic, cicatrizant, and anti-inflammatory properties (Nogueira et al., 2004; Oliveira et al., 2007).

The *S. marginata* Casar. species is a climbing plant popularly known as “cipó-uva” or “cipó-timbó”; the plant species is used in folk medicine in the form of infusion or juice for the treatment of stomach pains (Arruda et al., 2009). The species is considered native to Brazil, Paraguay, Bolivia and Argentina. In Brazil, the *S. marginata* Casar. species has been found to be present in deciduous forests, floodable fields, and in the Chaquenha region of Mato Grosso do Sul, as well as in 18 forest fragments in the northwest of the State of São Paulo, and in the dense forest of the State of Pernambuco (Rodal and Nascimento, 2002; Moreira et al., 2013; Sprengel-Lima and Rezende, 2013).

Previous phytochemical studies conducted on the leaf extracts of the plant species mentioned above have shown that *B. intermedia* has phenolic acids, oligomeric proanthocyanidins, and flavonoids as its main constituents (Pereira et al., 2015; Fraige et al., 2018; Mannochio-Russo et al., 2020) and *S. marginata* has flavonoids, oligomeric proanthocyanidins and saponins as its main constituents (Heredia-Vieira et al., 2015).

In view of the chemical and biological potential of *B. intermedia* and *S. marginata* species*,* conducting qualitative chemical analyses of the leaves of these plants, based on a seasonal approach, is essentially relevant in the sense that it enables one to verify if there is chemical variation in the profile of the species harvested at different times of the year due to the environmental factors evaluated related to the season (temperature, humidity, solar radiation and rainfall), apart from helping to identify the biological potential of the plant matrix investigated. In the present work, state-of-the-art tools in metabolic profiling and data analysis methods based on liquid chromatography LC-HRMS and NMR techniques were employed for the conduct of qualitative chemical analyses of the aforementioned plant species.

## Materials and Methods

### Chemicals

Methanol and formic acid (LC-MS grade) were obtained from Merck (Darmstadt, Germany) and ultrapurified water (Millipore^®^, United States) were used as mobile-phase components.

### Plant Material

*B. intermedia* leaves were harvested in the Municipal Botanical Garden of Bauru (JBMB), Bauru, state of São Paulo, Brazil (22°20′36″S and 49°01′02″W, altitude 546 m). The voucher specimens were deposited at Herbarium JBMB under number JBMB 00013 and responsibility of Dr. Viviane Camila de Oliveira.

*S. marginata* leaves were harvested in a fragment of Cerrado located in Santa Madalena Farm, Dourados, Mato Grosso do Sul state, Brazil (22°08′05″S and 55°08′17″W, altitude of 452 m). The voucher specimens were deposited at the DDMS Herbarium, of the Federal Universiy of Grande Dourados (UFGD), under number 41054 and responsibility of Dr Emerson Silva.

The harvesting of the plant was always carried out at the same period between 9 and 10 am for two consecutive years (2017 and 2018) to study the seasonal variability of their secondary metabolites. The detailed data for each harvest are described in Supplementary Table S1.

The harvested data were added in the SisGen platform (National System of Management of Genetic Heritage and Associated Traditional Knowledge) as genetic patrimony with registration number A3476AF. For the transfer of material between São Paulo State University—UNESP and the University of Strathclyde, all samples were prepared in accordance with the Brazilian laws for access and shipment of genetic heritage material. The R0418CB shipment number was issued by SisGen and under authorization from the Genetic Heritage Management Council (CCGEN).

### Hydroethanolic Extracts Preparation

The plant material (*B. intermedia* and *S. marginata* leaves) was washed and dried in an oven with air circulation at 40°C. The dry material was ground into an analytical mill (model IKA A11 basic). For the analytical analysis, 100 mg of powder was extracted with 1 ml of EtOH/H_2_O 7:3 (v/v) in an ultrasound bath, three times for 20 min. After that, the resulting material was centrifuged at 13,000 rpm and the supernatant was filtered through a Millex^®^ PTFE filter (0.22 μm, 25 mm). The extracted liquid was concentrated at a temperature below 40°C until removal of the organic solvent. The hydroethanolic extracts were frozen and lyophilized in a vacuum freeze dryer (BentchTop Pro SP Scientific) at 200 mT for 48 h at −60°C.

### Climate Data

The climatic data for all the harvests of each plant were provided by the Meteorological Data Storage Section (SADMET) of the National Institute of Meteorology (INMET). The climatic data were temperature (°C), humidity (%), solar radiation (kJ/m^2^) and rainfall (mm) for the years 2017 and 2018 provided in Supplementary Tables S2 and 3.

### UHPLC-(ESI)-HRMS Analysis

The chromatographic chemical profile analysis of the extracts of each species was performed by UHPLC-(ESI)-HRMS Thermo Scientific^®^ Accela using Thermo Scientific^®^ UHPLC Accela system coupled to an Exactive-Orbitrap high-resolution mass spectrometer Thermo Scientific^®^. To perform the analysis, the samples were suspended in methanol at a concentration of 1.0 mg/ml and filtered with Millex^®^ PTFE filter (0.22 μm, 25 mm). LC separations were conducted through a C-18 column (ACE, 75 mm, id 3.0 mm, 5 μm). The injection volume and flow rate applied were 10 and 300 μL/min, respectively. For sample elution, a linear gradient with mobile phase composed of water (solvent A) and methanol (solvent B) both acidified with 0.1% formic acid, from 5 to 100% (B) was employed for 45 min. The acquisition range was *m/z* 150–2000 in both negative and positive ionization modes. The spray voltage applied was 4.5 kV for positive mode and 4.0 kV for negative mode and capillary temperature set at 280°C. The mass spectra were obtained and processed in Xcalibur software (version 3.0).

### NMR Analysis

To conduct NMR analysis, samples were prepared by dissolving each sample in 650 μL of DMSO-*d*_*6*_ (Sigma Aldrich^®^) to obtain the concentration of 5 mg/ml then transferred to 5 mm 7″ NMR tubes. *J*-resolved and proton (^1^H NMR) experiments were performed using a Bruker^®^ AVIII HD 500 (11.7 T) and the spectra were processed using MestReNova x64 software (version 14.1.2).

The data acquisition followed the parameters used in the previous work developed by Sampaio et al. (2016) using 16 scans in the pre-saturation pulse sequence for one-dimensional (1D) proton spectra (^1^H NMR) and 32 scans and 64 increments per scan for two-dimensional (2D) ^1^H−^1^H *J*-resolved (*J-*res) NMR spectra; data points widths of 3.56 kHz for F2 (chemical shift axis) and 50 Hz for F1 (spin-spin coupling constant axis). The solvent signal was suppressed by the selective pre-saturation method.

### Data Processing

The UHPLC-(ESI)-HRMS data were converted to mzML format in the MSConvert (ProteoWizard) software using the filter Peak Picking with the algorithm vendor checked. mzML files were processed in MZmine2 v.2.53 (http://mzmine.sourceforge.net/) (Katajamaa et al., 2006; Pluskal et al., 2010) using the following parameters: mass detector, Centroid and MS1 noise level, 1 × 10^3^. For the construction of chromatogram, the parameters setting conditions were used: min time spam of 0.2 min; min height intensity of 1 × 10^4^ and mass tolerance of 0.001 m*/z* or 5.0 ppm; for the deconvolution, the algorithm local minimum search was chosen using: chromatographic threshold of 5%; search miminum in RT range of 0.4 min; minimum absolute height of 1 × 10^4^; min ratio of peak top/edge and peak duration range of 0.2–5 min. For data processing was applied deisotoping, filtering, alignment and gap filling steps. The identification of adducts and complexes were carried out. The molecular formulas were calculated by the Formula Prediction for both ionization modes ([M+H]^+^ and [M-H]^−^) within the mass tolerance window 0.001 m*/z* or 5.0 ppm and applying the element counts (C, H, N, O, P, S) from the detected *m/z* value considering all the heuristic rules (elemental ratios, RDBE restrictions and isotope pattern filter) (Pluskal et al., 2012). The output data (peak areas, exact mass, molecular formula and retention times) of each sample were exported as a CSV file and uploaded into an in-house Excel macro with the Dictionary of Natural Products (DNP) database for peak dereplication (Macintyre et al., 2014). A blank solvent was analyzed together with the extracts by LC-HRMS during data processing. Therefore, by macro Excel, the peaks from the blank were extracted and removed, applying an algorithm based on the intensity ratio of *m/z* between the samples and the blank.

The NMR data were processed using MestReNova x64 software version 14.1.2 (Mestrelab Research S.L.^®^). First, for processing the 2D *J*-res spectra the T1 noise reduction, 45° tilt and symmetrization by *J*-res sensitivity enhancement parameters were used; and at last, spectra in the one-dimensional projection were extracted.

The ^1^H NMR and *J*-res projection spectra were previously stacked and then pre-processing according the following steps: baseline correction with the Whittaker Smoother option; apodization with Gaussian function of 1 GB [Hz]; normalization by the largest peak and smoothing Savitzky–Golay method. The processed NMR data were prepared using bin width of 0.04 ppm and the bin intensities method was average sum. Afterward, the spectral data were saved with the peak intensities. Chemical shifts (*δ*) values were established between 0.0 and 10.0 ppm for *S. marginata* samples data and 0.0–13.0 ppm for *B. intermedia*.

The data fusion method was performed for the concatenation of the processed MS and NMR data (Forshed et al., 2006; Sampaio et al., 2016). The data were pre-processed and organized in Excel^®^ being MS and NMR data divided into two blocks. Each block were scaled according to the following procedure:

1) The standard deviation (SD) calculation of all the corresponding peak areas of each *m/z*;
2) The sum of all SD and then
3) The division of the areas of the original peaks by the sum of SD. MS and NMR scaled data were merged in a single data matrix which undergone to multivariate analysis.

### Multivariate Analysis

The processed MS, NMR and MS-NMR fused data were submitted to multivariate statistical analyses to study the metabolites variation related to the climate data of each plant species *B. intermedia* and *S. marginata*. The data obtained from the analysis of the extracts were classified as chemical variables (primary variables ID) and the climatic data as environmental variables (secondary variables ID). The set of variables were scaled using the Pareto algorithm in the SIMCA-P software v. 15.0 (Umetrics^®^, Sweden). Partial Least Squares Discriminant Analysis regression (PLS-DA) and Orthogonal Partial Least Squares Discriminant Analysis (OPLS-DA) were performed with at least three independent replicates for each season. Afterward, the model was validated by the permutation tests (repeated 100 times). The model fitting was given by parameter R2X and the expected variation by Q2X.

The correlation and differences in the metabolite profile for the seasons of each plant were screened by the PLS-DA loadings plot. To better interpret a large number of responses, the chemical variables were classified according to their significance to the model by the parameter variable importance on projection (VIP). The 15 variables with the highest VIPs were selected for further analysis. Thereafter, the false discovery rate (FDR) method was applied as follows: ranking the *p*-values of the variables from low to high, multiplying each *p*-value by the number of variables (*N* = 15), and dividing by their order of rank (Benjamini and Hochberg, 1995; Wang et al., 2013). Finally, the resulting FDR were adjusted considering values ≤0.05 to be significant.

The web-based platform MetaboAnalyst 4.0 (https://www.metaboanalyst.ca/) (Chong et al., 2018) was also used in the PLS-DA and hierarchical analysis. For the purposes of processing, the MS-NMR fusion data was filtered by the mean intensity value (Hackstadt and Hess, 2009), the samples were normalized by sum and the set of variables were scaled using the Pareto algorithm. The normalized output data were exported as a CSV file and analyzed using GraphPad Prism 8.4.3 (GraphPad Software, San Diego, CA, United States). The statistical significance between results obtained for the seasonal groups were analyzed using one-way ANOVA followed by Tukey’s test. All results are presented as mean ± standard deviation (SD).

## Results

### Metabolite Profiling Annotation by NMR and MS

A meticulous analysis based on the negative and positive ESI-HRMS spectral data, ^1^H NMR spectra, as well as 2D *J*-res spectra, allowed the annotation of the compounds present in the plant species *B. intermedia* and *S. marginata* extracts. MS and NMR features assignments were founded according to data previously reported in the literature for representative classes of metabolites or chemical markers for each genus and/or family studied. The comprehensive annotation of the compounds for each plant species are provided in the Supplementary Tables S4 and 5 (based on an accurate mass and molecular formulas). Supplementary Figures S1 to 8 shows the representative LC-HRMS chromatograms, the ^1^H NMR spectra, 1D *J*-res NMR projection spectra and the 2D *J-*res NMR for each plant extract.

Due to the complexity of the plant matrices, the ^1^H NMR spectra of the analyzed extracts show critical overlapping signals mainly as a result of the inherent signal splitting and occurrence of multiplet patterns. Therefore, 2D *J-*res NMR experiments proved to be a suitable choice to solve the problem of signal congestion. By projecting the *J-*res spectrum onto the chemical shift axis, the degree of spectral complexity was reduced, and the spectral resolution was increased (Supplementary Figures S5 to 8).

Considering the NMR identification, three main chemical shift regions were clearly distinguished in the spectra of *B. intermedia* and *S. marginata* samples, with peaks in the aliphatic (0.50–2.00 ppm), sugar and organic acids (3.00–6.00 ppm) and aromatic region (6.00–9.00 ppm) (Supplementary Figures S5 to 8). Regarding the carbohydrate portion of the compounds, the region of 4.00–6.00 ppm can be assigned to the signals of the anomeric protons of the sugars. In addition, the multiplicity and chemical shifts of these signals obtained in the 1D and 2D *J*-res spectra helped to propose the nature of the glycosides.

ESI-HRMS data annotation in positive and/or negative modes allowed to distinguish several classes of secondary metabolites in the three plant species.

*B. intermedia* extracts exhibited signals of cinnamic acids and derivatives (e.g., *m/z* 163.0396 [M-H]^−^, *m/z* 199.0605 [M+H]^+^, *m/z* 339.1078 [M+H]^+^, *m/z* 355.1025 [M+H]^+^, *m/z* 369.1183 [M+H]^+^ and *m/z* 531.1503 [M+H]^+^), galloylquinic acids (e.g., *m/z* 345.0819 [M+H]^+^, *m/z* 497.0930 [M+H]^+^, *m/z* 649.1040 [M+H]^+^ and *m/z* 801.1151 [M+H]^+^), galloylshikimic acids (e.g., *m/z* 327.0713 [M+H]^+^, *m/z* 479.0825 [M+H]^+^ and *m/z* 631.0937 [M+H]^+^), proanthocyanidins [e.g., monomer (*m/z* 291.0864 [M+H]^+^), dimer (*m/z* 577.1351 [M+H]^+^, tetramer (*m/z* 1153.2609 [M+H]^+^)], flavonoids derived from quercetin, such as monoglycosylated flavonoids (*m/z* 435.0928 [M+H]^+^, *m/z* 449.1085 [M+H]^+^ and *m/z* 465.1035 [M+H]^+^), diglycosylated flavonoids (*m/z* 581.1511 [M+H]^+^, *m/z* 597.1456 [M+H]^+^ and *m/z* 611.1617 [M+H]^+^), triglycosylated flavonoids (*m/z* 757.2195 [M+H]^+^) and galloyl flavonoids (*m/z* 587.1041 [M+H]^+^, *m/z* 601.1197 [M+H]^+^ and *m/z* 617.1147 [M+H]^+^), pentacyclic triterpenes with lupane and oleanane structures (e.g., *m/z* 427.3938 [M+H]^+^, *m/z* 455.3546 [M-H]^−^, *m/z* 487.3422 [M+H]^+^ and *m/z* 489.3579 [M+H]^+^), and other compounds (Supplementary Table S4).

*S. marginata* was characterized by having phenolic acids (e.g., *m/z* 153.0186 [M-H]^−^ and *m/z* 315.0724 [M-H]^−^), cinnamic acids (e.g., *m/z* 179.0345 [M-H]^−^, *m/z* 199.0600 [M-H]^−^ and *m/z* 343.0810 [M+H]^+^), triterpenic saponins derived from oleanolic acid (e.g., *m/z* 733.4550 [M-H]^−^, *m/z* 865.4971 [M-H]^−^, *m/z* 895.5082 [M-H]^−^, *m/z* 911.5031 [M-H]^−^, *m/z* 941.5128 [M-H]^−^, *m/z* 1011.5554 [M-H]^−^, *m/z* 1027.5503 [M-H]^−^, *m/z* 1043.5452 [M-H]^−^, *m/z* 1057.5609 [M-H]^−^, and so on), B-type proanthocyanidins [e.g., monomer (*m/z* 291.0860 [M+H]^+^), dimer (*m/z* 579.1500 [M+H]^+^), trimer (*m/z* 867.2117 [M+H]^+^) and tetramer (*m/z* 1155.2719 [M+H]^+^)] and A-type proanthocyanidins [e.g., dimer (*m/z* 577.1343 [M+H]^+^), trimer *m/z* 863.1810 [M+H]^+^), tetramer (*m/z* 1153.2602 [M+H]^+^) and pentamer (*m/z* 1441.3228 [M+H]^+^)], flavonoids glycosylated including *C*-glycosylated flavones (e.g., *m/z* 403.1023 [M+H]^+^, *m/z* 417.1177 [M+H]^+^ and *m/z* 419.0973 [M+H]^+^), *m/z* 597.1451 [M+H]^+^ and *m/z* 609.1479 [M-H]^−^), *C*,*O*-glycosylated flavones (e.g., *m/z* 551.1394 [M+H]^+^, *m/z* 561.1603 [M+H]^+^, *m/z* 563.1761 [M+H]^+^, *m/z* 565.1550 [M+H]^+^, *m/z* 577.1551 [M+H]^+^, *m/z* 579.1707 [M+H]^+^ and *m/z* 591.1708 [M+H]^+^), and *O*-glycosylated flavonols (e.g., *m/z* 433.1129 [M+H]^+^, *m/z* 465.1027 [M+H]^+^ and *m/z* 595.1658 [M+H]^+^), among other compounds (Supplementary Table S5).

### Seasonality Assessment From MS-NMR Fused Data

**FIGURE 1:**
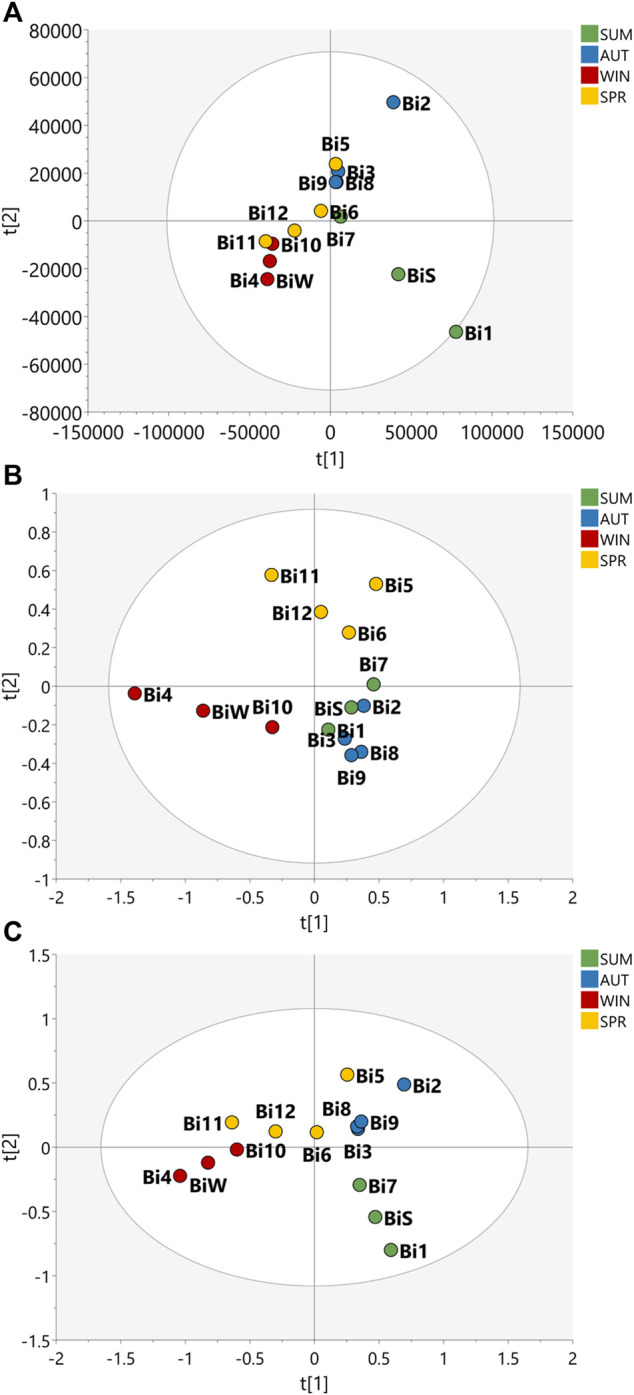
Partial Least Squares Discriminant Analysis (PLS-DA) of the data acquired for the samples of the seasonal harvests of *B. intermedia*. PLS-DA score scatter plot of **(A)** HRMS, **(B)** *J*-res NMR and **(C)** MS-NMR fused data of *B. intermedia* extracts of summer (SUM), autumn (AUT), winter (WIN), and spring (SPR) harvests.

**FIGURE 2:**
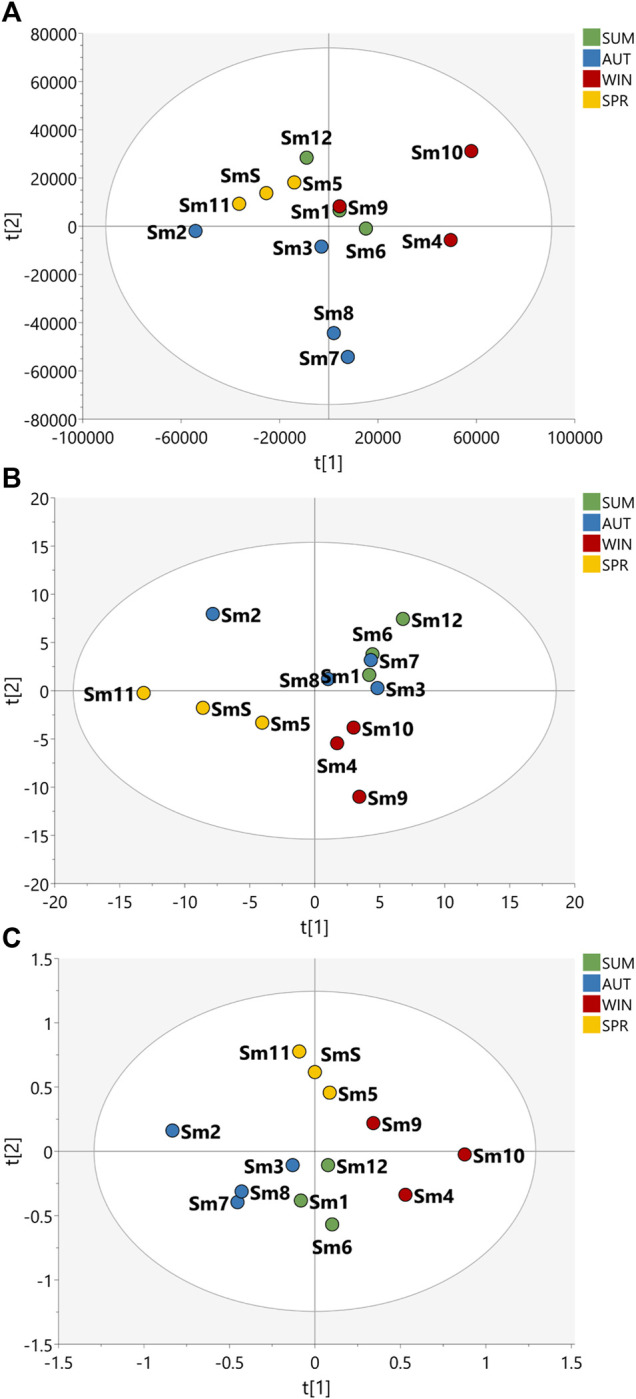
Partial Least Squares Discriminant Analysis (PLS-DA) of the data acquired for the samples of the seasonal harvests of *S. marginata*. PLS-DA score scatter plot of **(A)** HRMS, **(B)** *J*-res NMR and **(C)** MS-NMR fused data of *S. marginata* extracts of summer (SUM), autumn (AUT), winter (WIN), and spring (SPR) harvests.

Multivariate analysis of HRMS and 2D *J*-res NMR data were evaluated to assess the seasonality of the *B. intermedia* and *S. marginata* harvests. MS and NMR data were evaluated separately and concatenated (Figures 1, 2).

The PLS-DA plot in Figure 1 shows that the *B. intermedia* harvests had a significant seasonal effect, with separation into distinct groups, especially for summer and winter samples. In Figure 1A, clustering was performed according to HRMS data and it is apparent that the spring samples presented an intermediate MS profile between winter and autumn seasons; in Figure 1B, for the *J*-res data, summer and autumn had more similarity in their NMR profiles which contributed to the clustering of these samples and finally in Figure 1C, the concatenation of HRMS and *J*-res data resulted in an improved clustering of the harvests samples from the same season and more clearly represented the differentiation in the sets of seasons according to the variation in their features.

With regard to *S. marginata* harvests, seasonality appeared not to have a significant influence on the differentiation of samples, demonstrating only a slight distinction of samples harvested during spring in comparison to the other seasonal groups. For the HRMS dataset in Figure 2A, the samples are quite scattered, with no clustering trends within seasons; in Figure 2B, the *J*-res data contributed to the separation of spring and winter into two distinct groups, while the autumn and summer seasons had closer clustering, and then finally, in Figure 2C, with the concatenated HRMS and *J*-res data, it can be observed that the samples are more scattered, that is, the sets of each season do not cluster tightly, moreover, summer showed to present a chemical profile with intermediate features between autumn and winter seasons.

**FIGURE 3:**
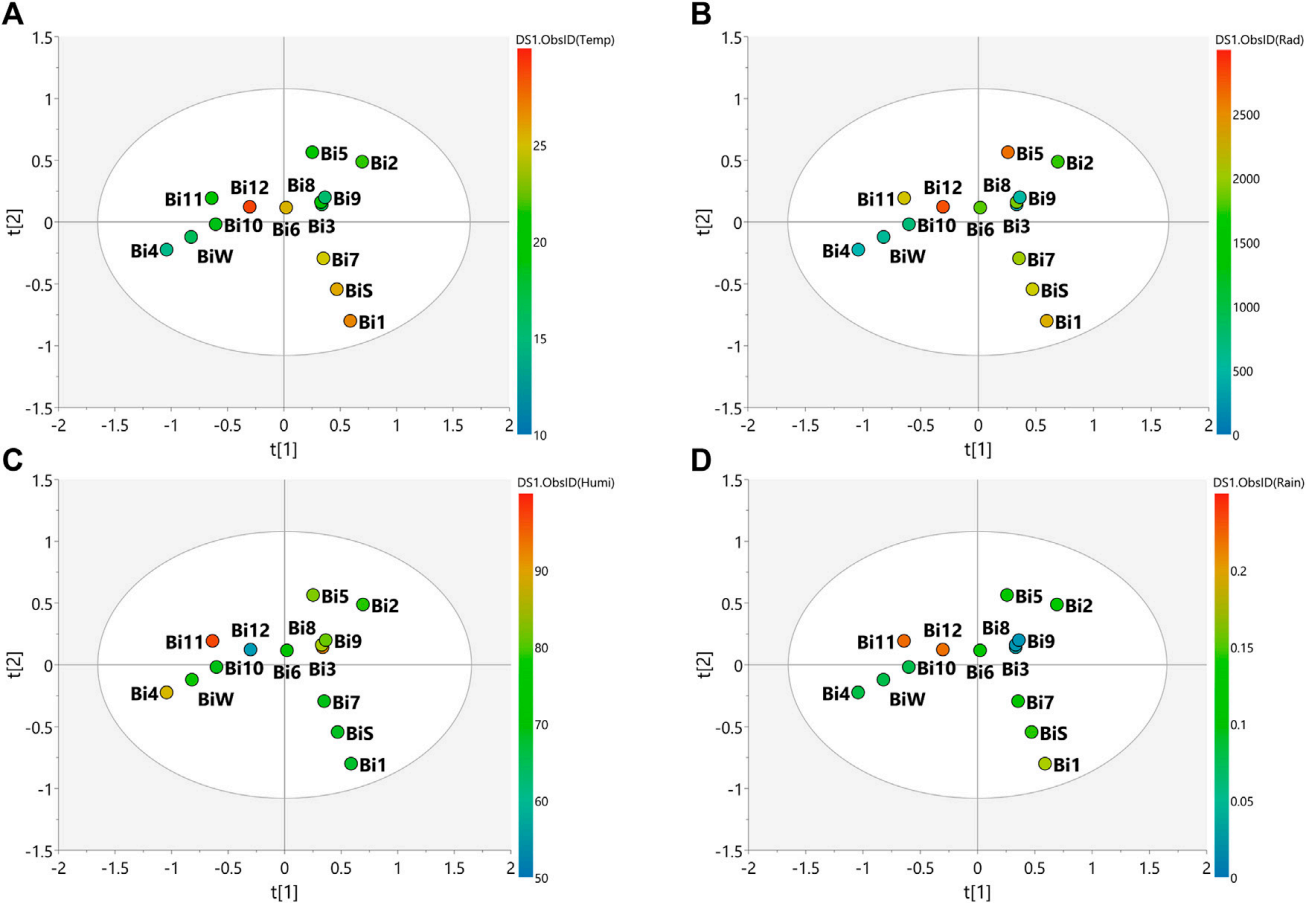
PLS-DA score scatter plots of the MS-NMR fused data of *B. intermedia* seasonal harvests and the correlation with the environmental factors. Samples are categorized according to the values of observations for environmental factors. **(A)** Temp = temperature (°C); **(B)** Rad = Solar radiation (kJ/m^2^); **(C)** Humi: relative humidity (%) and **(D)** Rain = rainfall (mm). Sample seasons: Summer (Bi1, Bi7 and BiS); Autumn (Bi2, Bi3, Bi8 and Bi9); Winter (Bi4, Bi10 and BiW) and Spring (Bi5, Bi6, Bi11 and Bi12).

**FIGURE 4:**
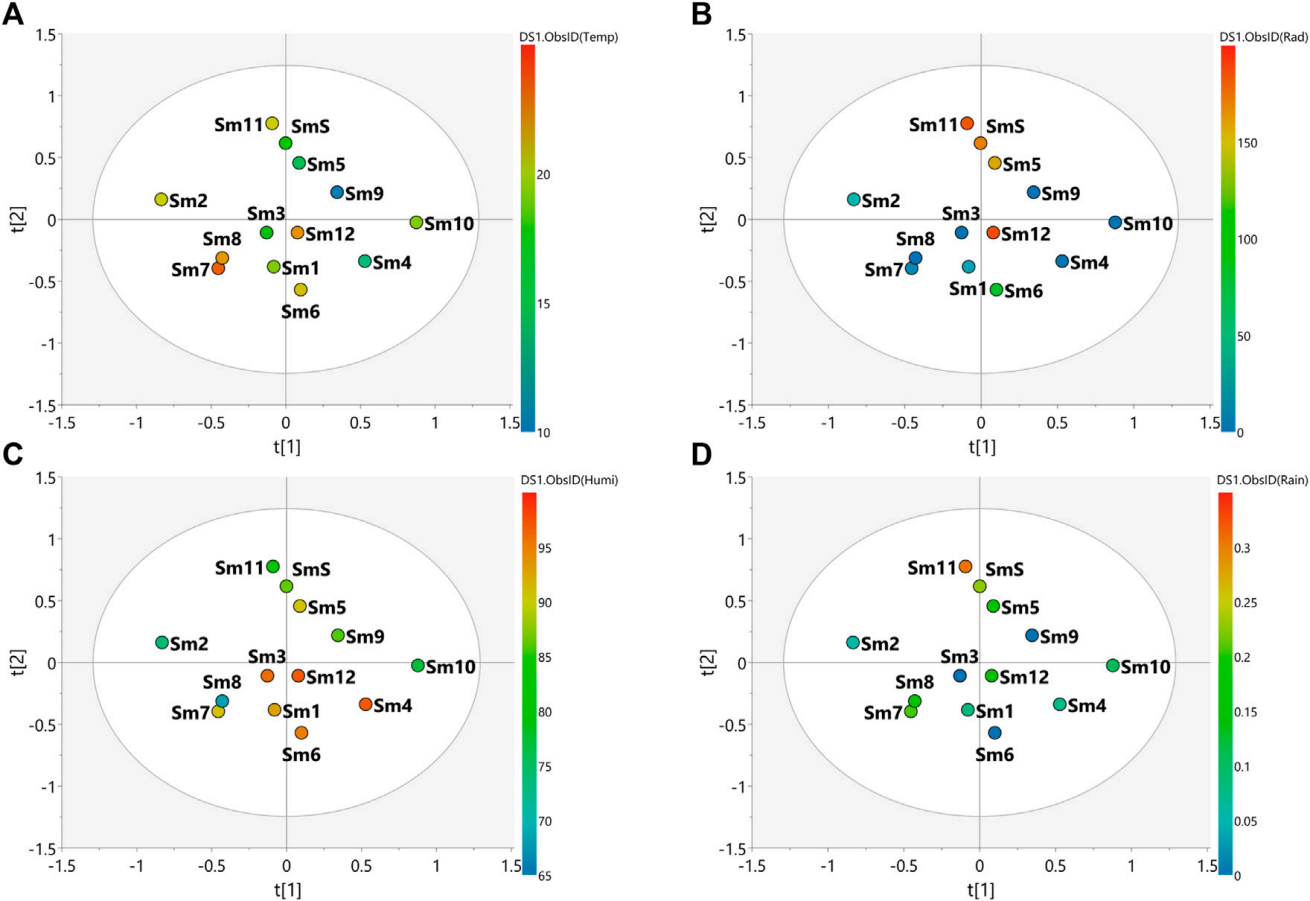
PLS-DA score scatter plots of the MS-NMR fused data of *S. marginata* seasonal harvests and the correlation with the environmental factors. Samples are categorized according to the values of observations for environmental factors. **(A)** Temp = temperature (°C); **(B)** Rad = Solar radiation (kJ/m^2^); **(C)** Humi: relative humidity (%) and **(D)** Rain = rainfall (mm). Sample seasons: Summer (Sm1, Sm6 and Sm12); Autumn (Sm2, Sm3, Sm7 and Sm8); Winter (Sm4, Sm9 and Sm10) and Spring (Sm5, Sm11 and SmS).

The PLS-DA score scatter presented in Figures 3, 4 for the plant species samples, correlates the MS-NMR fused data of seasonal harvests plotted against the environmental factors. We choose to categorize the plant species according to the values of observations for main environmental factors observed in the region of the harvests (Temperature, Solar radiation, Relative humidity and Rainfall). Average values of weather characteristics of the local where each plant species was harvested are shown Supplementary Tables S2 and 3.

In view of the weather characteristics of the local where *B. intermedia* was harvested (Supplementary Table S2), it is possible to notice a lower rainfall amount during autumn and winter, and a higher amount during spring (Figure 3D). In addition, spring and summer periods had the warmest temperatures (Figure 3A) and the most intense incidence of solar radiation (Figure 3B). Visual inspection of the grouping of samples and the climate observations in the plot of Figure 3 shows that the temperature is the climate variable which better explained the correlation between the samples, besides being the only variable that explained the correlation of the spring and winter samples and the influence on the chemical composition (Figure 3A). The samples harvested during winter (Bi4, Bi10 and BiW) proved to be the most homogeneous and are more tightly grouped, indicating minimal changes in the chemical profiles for the samples harvested in this season and this is due to the lower climatic variation during this period. Spring was the most heterogeneous season with the greatest dispersion of samples (Bi5, Bi6, Bi11, Bi12), indicating a greater variation in the chemical profiles of these samples. This season is intermediate between winter and summer and was influenced by the climatic conditions of late winter and early summer.

The main weather conditions for *S. marginata* harvests (Supplementary Table S3) indicate higher temperatures during the summer and autumn periods (Figure 4A), high humidity levels in the summer (Figure 4C) and higher rainfall amounts in the spring (Figure 4D). The spring season (Sm5, Sm11 and SmS) presented the most separated data in relation to the other groups, indicating differences in the chemical profile in relation to the other samples. In addition, the spring season showed the tightest clusters relative to the other seasons indicating minimal changes in chemical profiles for the samples harvested in this season. The autumn (Sm2, Sm3, Sm7 and Sm8), summer (Sm1, Sm6 and Sm12) and winter (Sm4, Sm9 and Sm10) samples were more scattered indicating a greater variation in the chemical profiles of these samples. Considering the environmental factors, it is more difficult to correlate the variation in chemical composition with the climate variables. In the PLS-DA plot, solar radiation incidence seems to be the variable with the greatest variation, especially with higher values for the spring season and lower values for autumn and winter, which may influence the chemical profile of these samples according to this environmental factor (Figure 4B).

To validate the model, OPLS-DA data analysis was used. For this purpose, the samples were separated into more scattered and more clustered groups. The summer season was the most scattered set of samples relative to the other season groups for *B. intermedia* and the spring season was most scattered for *S. marginata*. The Permutations plots (Supplementary Figure S9) show the model fit based on the R2 and Q2 values.

A total of 15 variables with the highest VIP scores were selected to validate the chemical features that are significant for group discrimination in the OPLS-DA model for the MS-NMR fused and HRMS data. Subsequently, after validation of the significant NMR regions contributing to seasonal group discrimination and putative annotation of functional groups and classes of compounds, the LC-HRMS data were analyzed for further insights into the annotation of the chemical structures of the discriminant compounds.

**FIGURE 5:**
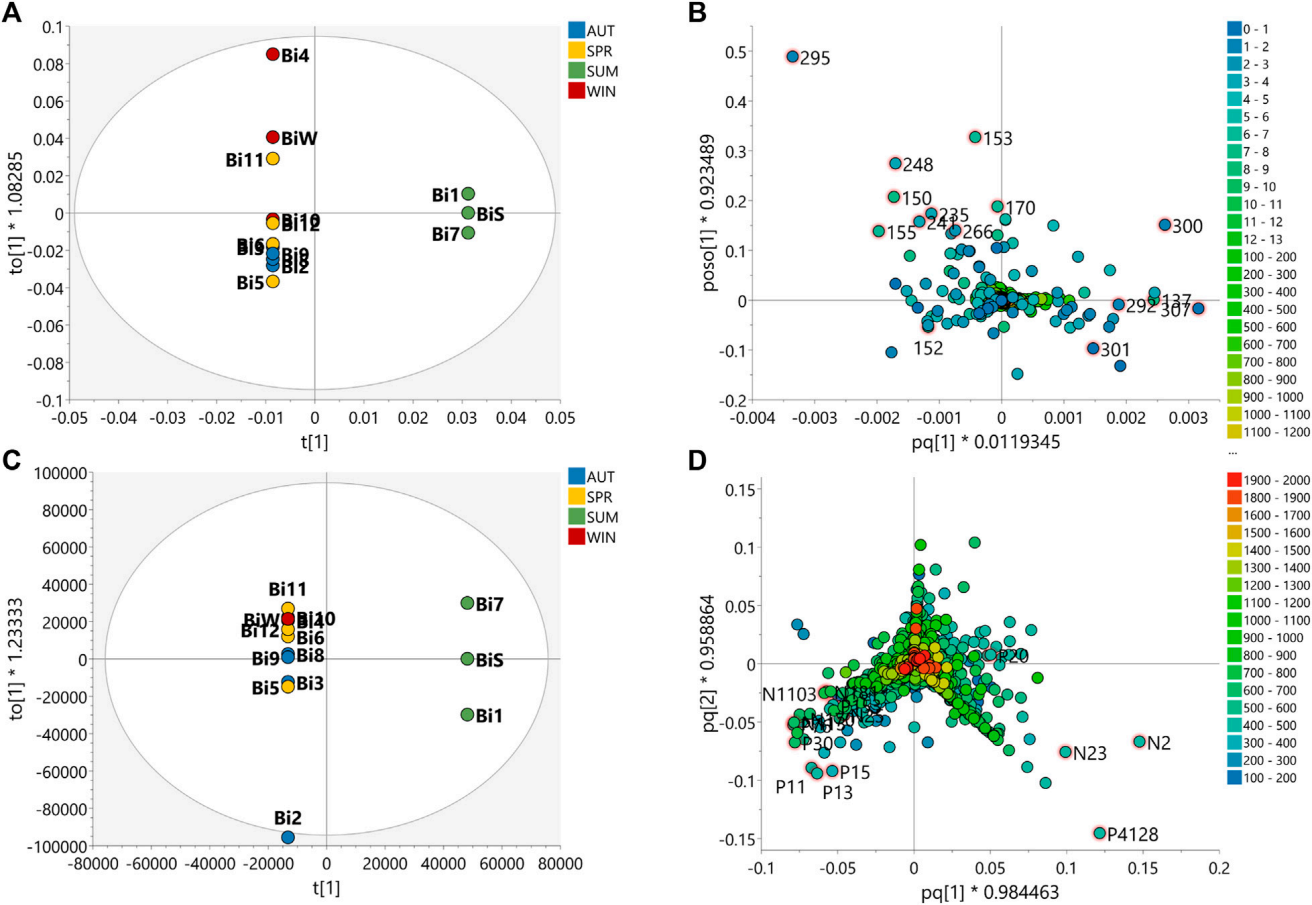
Multivariate analyses of the data of *B. intermedia* species for prediction of the seasonally discriminating metabolites. Orthogonal Partial Least Square-Discriminant Analysis (OPLS-DA) score scatter plot of **(A)** MS-NMR fused data and **(C)** HRMS data with samples categorized according to the harvest season. OPLS-DA loadings plots with highlighted features of the 15 VIP metabolites of **(B)** MS-NMR fused data and **(D)** HRMS data. More scattered samples: Summer (Bi1, Bi7 and BiS); Less scattered samples: Autumn (Bi2, Bi3, Bi8 and Bi9); Winter (Bi4, Bi10 and BiW) and Spring (Bi5*,* Bi6, Bi11 and Bi12).

**FIGURE 6:**
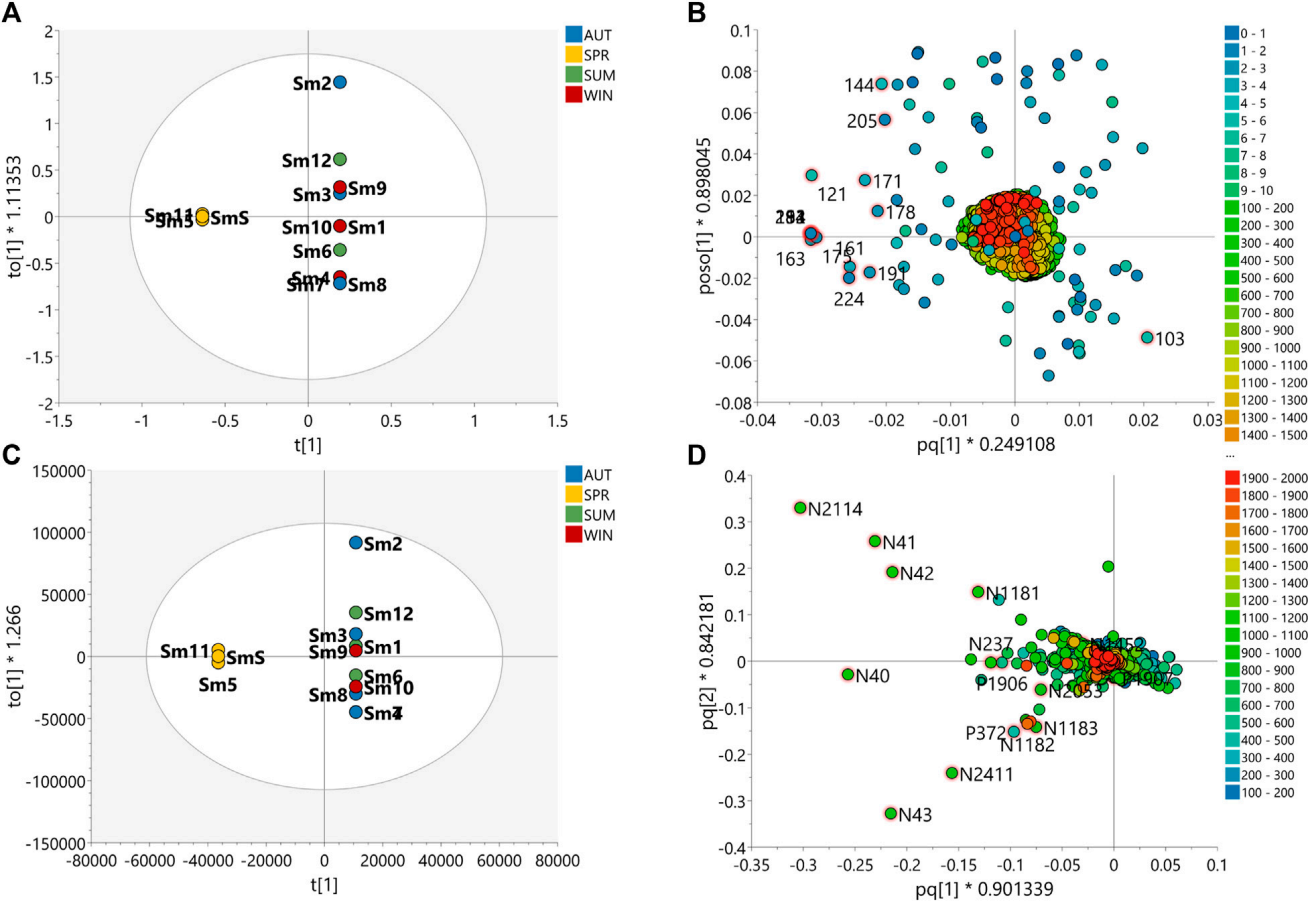
Multivariate analyses of the data of *S. marginata* species for prediction of the seasonally discriminating metabolites. Orthogonal Partial Least Square-Discriminant Analysis (OPLS-DA) score scatter plot of **(A)** MS-NMR fused data and **(C)** HRMS data with samples categorized according to the harvest season. OPLS-DA loadings plots with highlighted features of the 15 VIP metabolites of **(B)** MS-NMR fused data and **(D)** HRMS data. More scattered samples: Spring (Sm5, Sm11 and SmS); Less scattered samples: Summer (Sm1, Sm6 and Sm12); Autumn (Sm2, Sm3, Sm7 and Sm8); Winter (Sm4, Sm9 and Sm10) and Spring (Sm5, Sm11 and SmS).

Figures 5, 6 show the scatter plots of the OPLS-DA score and the selected variables highlighted in the loading plots of the MS-NMR fused data and HRMS data, for *B. intermedia* and *S. marginata*, respectively. Supplementary Tables S6 and 7 show the adjusted *p*-values and FDR estimation for each discriminant feature of the MS-NMR fused data and Supplementary Figures S10 and 11 show the representative 1H NMR and 2D *J*-res NMR spectra with the top 15 peak signals according to the VIP scores. Supplementary Tables S8 and 9 show the same analysis for the discriminant metabolites found with respect to the results obtained from the MS data. For the discriminating metabolites, the chemical formula was predicted from the exact mass, the most probable ring double bond formula (RDB) was calculated, and the compounds were annotated according to the hits in the DNP database. In addition, box-and-wisker plots in Supplementary Figures S12 and 13 show the relative distribution of the discriminating metabolites according to seasonal harvest of *B. intermedia* and *S. marginata* samples, respectively.

OPLS-DA loadings plot for *B. intermedia*, show the selected VIP features, highlighting signals of chemical shifts in the aliphatic proton region (0.50–5.00 ppm) and aromatic region (6.00–8.00 ppm), and also molecular weights between 400 and 900 Da correlated to summer and autumn seasons (Figure 5B). The chemical shift signals at *δ* 7.53 (**137**), 6.90 (**153**), 1.35 (**292**), 2.39 (**266**), 7.04 (**150**), 3.12 (**248**), 3.36 (**241**), 6.21 (**170**), 3.62 (**235**), 0.77 (**307**) and 1.24 (**295**) presented FDR values lower than 0.05, representing the true positive annotation and indicating the discriminating metabolites associated with seasonal harvests (Supplementary Table S6). The signals at *δ* 7.53 (d, *J* = 2.3 Hz) and *δ* 6.21 (d, *J* = 2.1 Hz) indicate *meta*-couplings corresponding to the protons of the B and A rings of a flavonoid, respectively, which are consistent with the quercetin aglycone, while the signals at *δ* 3.12, *δ* 3.36 and *δ* 3.62 are characteristic of the protons of a saccharide unit, suggesting the presence of a glycosylated flavonoid (Harborne, 1994). The singlets at *δ* 7.04 and *δ* 6.90 were assigned to galloyl substituents, which may be attached to the structure of sugars or quinic acid by esterification of hydroxyl groups (Sannomiya et al., 2007). The chemical shifts of the shielded signals in the range *δ* 0.77–1.35 suggest methyl and methylene protons of triterpenoid compounds (Higuchi et al., 2008). Considering the loadings plot for the discriminant metabolites based on MS data (Supplementary Figure S5D), the flavonoids were annotated as quercetin-*O*-hexose (**P13**) and quercetin-*O*-(*O*-galloyl)-hexose (**N25**), the galoyl quinic acid derivatives as di-*O*-galloylquinic acid (**P76**) and tri-*O*-galloylquinic acid (**N113**) and the triterpenes as betulinic acid (**N2**), oleanolic acid (**N23**), *β*-amyrin (**P4128**) and 3-oxo**-**olean-12-en-28-al (**P22**) (Supplementary Table S8 and Figure S7). In Supplementary Figure S12, it is possible to notice a progressive increase in the concentration of phenolic compounds, such as flavonoids and galloylquinic acid derivatives, since summer until reaching maximum values in winter for *B. intermedia* harvests. On the other hand, the signals related to triterpenes show that these compounds presented higher concentration during the summer and minimum values during the winter.

**FIGURE 7:**
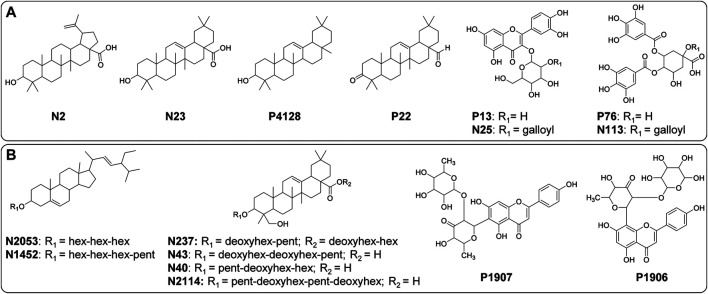
Structures of significant metabolites (FDR ≤ 0.05) for the differentiation of (**A**) *B. intermedia* (**N2**, **N23**, **P4128**, **P22**, **P13**, **N25**, **P76** and **N113**) and (**B**) *S. marginata* (**N2053**, **N1452**, **N237**, **N43**, **N40**, **N2114**, **P1907** and **P1906**) harvests. Annotation of the metabolites is provided in the Supplementary Tables 7 and 9.

OPLS-DA loadings of the fused data for *S. marginata* shows compounds with high molecular weight (colored in red) associated with typical chemical shifts of sugar and methylene protons (colored in blue) correlated with spring season (Figure 6B). The chemical shift signals at *δ* 3.99 (**151**), 3.52 (**163**), 2.72 (**182**), 2.27 (**194**), 1.49 (**213**), 5.19 (**121**), 3.03 (**175**), 1.08 (**224**), 3.58 (**161**) and 3.18 (**171**) were significant (FDR ≤0.05) for the differentiation of the seasons (Supplementary Table S7). The signals with chemical shifts in the range of *δ* 1.82–3.18 suggest methylene protons of sapogenins portions of saponin molecules. Analyzing the loadings plot and the annotation of the discriminating metabolites considering the MS data (Figure 6D), it was obtained that the steroidal saponins (**N2053** and **N1452**), triterpenic saponins with hederagenin as aglycone (**N237**, **P1907**, **N43**, **N40** and **N2114**) and the flavones cassiaocidentalin A (**P1907**) and tetrastigma B (**P1906**) are the compounds that contribute the most to the separation of the samples (Supplementary Table S9 and Figure S7). Samples harvested during spring had the highest amount of saponin, while winter samples showed the lowest amount. Flavones, on the other hand, had higher concentrations during winter, with a continuous increasing variation from summer to winter, followed by a decreasing in the concentration in the spring season (Supplementary Figure S13).

## Discussion

The analyses conducted for each set of samples using LC-HRMS and NMR showed that the extracts prepared from the leaves of the plant species *B. intermedia* and *S. marginata* had a comparable metabolite profile. Visual inspection of these data showed a variation in the intensity of the metabolites as a result of different harvest periods.

The computational analysis approach employed for the pre-processing of the untargeted LC-MS data was found to be very useful in terms of obtaining relevant information regarding the peak areas with a more accurate estimation of the relative ions abundance. Based on the application of the computational analysis approach, the present study was able to deconvolve the co-eluted peak signals and predict the molecular formulas of individual features of the metabolites with a high degree of accuracy. The application of chromatographic analysis promoted a good separation of the main groups of metabolites and the electrospray ionization technique (ESI) led to the unequivocal formation of deprotonated and protonated molecules.

The dereplication of the ESI-HRMS data using the macro database containing the Natural Products Dictionary (DNP) allowed a rapid and effective annotation of 68 compounds in the extracts of *B. intermedia* and 81 compounds in the extracts of *S. marginata* (see Supplementary Tables S4, S5).

As mentioned previously in the results section, the metabolite profile of the hydroethanolic extracts of *B. intermedia* revealed that this plant is a rich source of cinnamic acids, phenolic acids derived from galloyl quinic and shikimic acid, proanthocyanidins, glycosylated flavonoids, triterpenes and other phenols (Supplementary Table S4). Glycosylated flavonoids were among the major classes of compounds found in the *B. intermedia* plant species. These compounds (glycosylated flavonoids) were characterized as quercetin derivatives which consisted of pentoses, deoxyhexoses and hexoses that are attached to the flavonoid aglycone by *O*-glycosidic linkages. The quinic and shikimic acid derivatives which contain galloyl as substituents were also among the major classes of compounds found in *B. intermedia*; these compounds contained between one to four gallic acid units per molecule (≈300–800 Da).

In the ^1^H and 2D *J*-res NMR spectra (Supplementary Figures S3, 5 and 6), the main signals were observed in the aromatic region (*δ* 5.70–8.00) and can be assigned to the aromatic rings of the derivatives compounds of gallic acid, quercetin and catechin. The signals at *δ* 7.53 (d, *J* = 2.3 Hz), *δ* 7.67 (dd, *J* = 2.3; 8.5 Hz) and *δ* 6.83 (d, *J* = 8.5 Hz) were assigned to the protons of the trisubstituted aromatic ring. Two doublets with chemical shifts at *δ* 6.41 (d, *J* = 2.1 Hz); 6.21 (d, *J* = 2.1 Hz) indicate a *meta*-coupling corresponding to the protons of the A-ring flavonoid, which are consistent with the quercetin aglycone (Harborne, 1994). Whereas, the A-ring protons of the catechin derivatives are more shielded than those assigned to quercetin, and therefore were assigned to the doublet signals at *δ* 5.71 (d, *J* = 2.1 Hz) and *δ* 5.90 (d, *J* = 2.1 Hz). The singlet at *δ* 7.03 is characteristic of the two protons of the gallic acid/galoyl group aromatic ring.

In addition, the signals at *δ* 5.37 (d, *J* = 7.7 Hz), 5.27 (d, *J* = 3.8 Hz) and 4.55 (d, *J* = 1.4 Hz) were assigned to the anomeric protons of the sugar units glucose, arabinose and rhamnose, respectively and thereby confirming the presence of glycosylated flavonoids (Supplementary Figures S3, 5 and 6). The anomeric configurations for the sugar units were deduced from their ^3^ *J*_H-1/H-2_ coupling constants. The value of *J* for the signal at *δ* 5.37 indicates that the glycoside has a *β* configuration (*J* > 5.0 Hz), while for signals at *δ* 5.27 and 4.55 the configuration was assigned as *α* (*J* = 0–5.0 Hz) (Agrawal, 1992).

The signals of the compounds of the triterpene class can be observed in the aliphatic region of the 1H and 2D *J*-res NMR spectra; the signals in the range of *δ* 0.50–3.50 can be attributed to the methyl and methylene protons and some signals in the region of *δ* 3.50–5.00 to the oxymethynic protons of the triterpene structure (Higuchi et al., 2008).

The extracts of *S. marginata* showed mainly exhibited compounds derived from saponins, glycosylated flavonoids, catechins and oligomeric proanthocyanidins (Supplementary Table S5). The detection of saponins in *S. marginata* in this study helped to confirm the findings reported in the literature which pointed out that saponins are the major class of compounds in this plant species (Heredia-Vieira et al., 2015). Precisely, the results of our study showed that triterpenic saponins were prevalent in the extracts of *S. marginata*; these compounds contained aglycones of oleanolic acid (olean-12-en-28-oic), hederagenin (3,23-dihydroxyolean-12-en-28-acid) and gypsogenin (3-hydroxy-23-oxo-olean-12-en-28-acid). The presence of glycosylated flavonoids was detected in three groups: *C*-glycosylated, *C,O*-glycosylated and *O*-glycosylated flavonoids (Supplementary Table S5). The compounds tetrastigma B (*m/z* 561.1603 [M+H]^+^, R_t_ = 9.89) and cassiaocidentalin A (*m/z* 561.1602 [M+H]^+^, R_t_ = 10.37) are examples of *C,O*-glycosylated flavonoids isomers with an interglycosidic linkage between a deoxyhexose and 6-dideoxyhexose sugars (Heredia-Vieira et al., 2015). Two types of proanthocyanidin compounds were found in *S. marginata*; these included B-type and A-type proanthocyanidins (Supplementary Table S5). B-type proanthocyanidins are characterized by a single interflavan linkage between monomeric units which can be formed by the equivalent units of (epi)catechin. A-type proanthocyanidins, on the other hand, contain double interflavan bonds.

The ^1^H and 2D *J*-res NMR spectra recorded for the *S. marginata* extracts showed intense signals in the aromatic proton region between 5.70 and 8.10 ppm and an aliphatic region between 0.50 and 5.60 ppm, indicating characteristic signals corresponding to compounds of the flavonoid and saponin classes, respectively (Supplementary Figures S4, 7 and 8).

As observed in the mass spectrometry analyses, *S. marginata* has mainly flavonoids derived from apigenin and catechin. The most intense signals observed in the spectrum at *δ* 8.03–7.95 (d, *J* = 9.1 Hz) and other signals at *δ* 7.00–6.96 (d, *J* = 9.1 Hz) in the aromatic region can be attributed to the *ortho* coupling of the H-2′/H-6′ and H-3′/H-5′ protons, respectively, characteristic of a *para*-substituted aromatic ring like those observed for the aglycone derivatives of apigenin. The singlet at *δ* 6.55 was assigned to H-8 when ring A is substituted at position 6 and the singlet at *δ* 6.23 was assigned to H-6 when ring A is substituted at position 8. These signals corroborate the proposal that *C*-glycosylated isomers are found with the glycoside attached at the C-6 or C-8 position of the aglycone. Regarding the glycosides attached to flavonoids, the signals at *δ* 5.36 (d, *J* = 10.0 Hz) and *δ* 5.00 (d, *J* = 10.4 Hz) are consistent with the anomeric protons of a 6-deoxy-ribo-hex-3-ulose sugar units. Also, the doublets in *δ* 1.41 (d, *J* = 6.0 Hz) and *δ* 1.31 (d, *J* = 6.1 Hz), were assigned to the methyl groups of this sugar unit. Therefore, the data presented corroborate the presence of the compounds tetrastigma B and cassiaocidentalin A (Heredia-Vieira et al., 2015).

The signals in the ^1^H and 2D *J*-res NMR spectra of double doublet at *δ* 7.29 (*J* = 8.3, 2.1 Hz), doublets with *meta* couplings at *δ* 7.07 (*J* = 2.3 Hz) and *δ* 7.03 (J = 2.5 Hz), doublets with *ortho* couplings *δ* 6.78 (*J* = 8.2 Hz), *δ* 6.74 (*J* = 8.3 Hz) and *δ* 6.68 (*J* = 8.2 Hz) and the singlets at *δ* 6.08 and *δ* 5.96 are some examples of signals which can be attributed to the rings of the (epi)catechin units of the proanthocyanidins (Kolodziej, 1992; Heredia-Vieira et al., 2015). Furthermore, the signals at *δ* 4.00–4.95 with coupling constants of 3.3–3.7 Hz are diagnostic features of the AB coupling systems of A-type proanthocyanidins (Jacques et al., 1974; Kolodziej, 1992; Lou et al., 1999).

The main signals observed for the saponins in the ^1^H and 2D *J*-res NMR spectra were the signals at *δ* 5.33 (s), *δ* 5.20 (d, *J* = 3.7 Hz), *δ* 5.18 (d, *J* = 3.5 Hz) and *δ* 4.30 (d, *J* = 6.4 Hz), regarding anomeric protons and the presence of singlets between *δ* 0.73 and 1.31, characteristic of methyl protons attached to the triterpenoid nucleus (Heredia-Vieira et al., 2015).

A number studies published in the literature have shown that these compounds found in each plant species exhibit a wide range of biological activities. Phenolic compounds such as phenolic acids, tannins and flavonoid derivatives found in the plant species have been shown to have good antioxidant properties, which are essentially useful for the prevention and treatment of various inflammatory diseases including gastric ulcer, diabetes and obesity (Salinas-Sánchez et al., 2017; Bakoyiannis et al., 2019; Ma et al., 2019). Inflammatory diseases are often associated with the production and release of pro-inflammatory mediators, free radicals, ROS, and DNA-reactive aldehydes from lipid peroxidation (Anderson et al., 2014; Otimenyin, 2018). Compounds with antioxidant capacity are able to capture free radicals and reduce their creation (i.e. the creation of free radicals), chelate transition metals, and inhibit some enzymes involved in oxidative processes (Huang et al., 2005; Biswas et al., 2017; Tungmunnithum et al., 2018). Some previous studies attributed the anti-inflammatory action of glycosylated flavonoids to their ability to decrease the production of pro-inflammatory mediators and biomarkers of oxidative stress, by suppressing or alleviating the progression of inflammation (Jiang et al., 2017; Fraige et al., 2018; Dantas-Medeiros et al., 2021).

Proanthocyanidins can also act as antioxidants; in fact, according to studies reported in the literature, the bioavailability of these compounds is higher for monomers and small oligomers, such as those found in the extracts of the plant species investigated here (Cos et al., 2004; Rauf et al., 2019). Among the derivatives of gallic acid, such as galloylquinic acid and gallotannins derivatives, the galloyl group is found to possess antioxidant properties; in fact, reports in the literature have shown that an increment in the number of galloyl groups increases the antioxidant activity of the compound (Baratto et al., 2003; Karas et al., 2017). Interestingly, some studies have reported that the addition of a galloyl group to the structure of flavonoids glycosides and proanthocynidins leads to an increase in radical scavenging activity compared to non-galloylated compounds (Karas et al., 2017).

Other compounds that deserve mentioning are triterpenes and saponins. Triterpenes have been shown to have anti-inflammatory, anticarcinogenic, antidiabetic, antitubercular, hepatoprotective, antimicrobial, antimycotic, analgesic, immunomodulatory, and cardiotonic activities (Higuchi et al., 2008; Agra et al., 2015; Dinday and Ghosh, 2020). Saponins have also been found to possess antifungal, molluscicidal, antibacterial, hemolytic, anti-inflammatory, antiparasitic, antitumor, cytotoxic, antiviral, insecticidal, among other biological properties (Ekabo et al., 1996; Sparg et al., 2004; Biswas and Dwivedi, 2019). Some studies have shown that some structural features of triterpenes and saponins are related to an increase in their bioactivities; these features include glycosylation, and/or side-chain methylation or hydroxylation (Zhang et al., 2007; Biswas and Dwivedi, 2019).

Triterpenes are hydrophobic structures while saponins exhibit a hydrophobic aglycone and hydrophilic sugar groups; these differences in the structures of these compounds mean that saponins, being less lipophilic, are more bioavailable than triterpenes. In addition, these characteristics of saponins structure with non-polar and polar portions, give them surface-active properties and therefore they are generally considered as haemolysing agents (Biswas and Dwivedi, 2019; Dinday and Ghosh, 2020).

Apart from their pharmacological properties, the specialized metabolites found in *B. intermedia* and *S. marginata* species have also been found to play important ecological roles and their chemically distinct groups act in different ways in the defense of these plants. As aforementioned, several studies published in the literature have described the chemical composition of *B. intermedia* and *S. marginata* species and have pointed out the various health benefits that these species bring to humans. However, knowledge is still very limited regarding the relationship between the chemical composition of these plant species and the variation observed in the metabolites when these species are harvested at different periods of the year. Thus, to have a better understanding of the influence of seasonality on the production of metabolites in *B. intermedia* and *S. marginata*, the HRMS and 2D *J*-res NMR data sets were evaluated using multivariate analysis.

Based on the PLS-DA plots (Figures 1, 2) it was possible to visualize the differences in terms of clustering between the samples according to the features used; this shows that MS and NMR contribute uniquely to the analysis of statistical metabolomics analysis of the plant species. In addition, one can say that the data obtained from *J*-res NMR analysis provided highly accurate information, as it shows a greater contribution to the correlation and differentiation of the samples when the data is concatenated.

The permutation plot (Supplementary Figure S9) showed that the regression line (in blue) of the Q2-points intersected the vertical axis below zero; this strongly indicates that the model is valid and not overfitted. This result also demonstrates that the model shows good correlation (of data) and a reliable degree of predictability.

Based on the application of variable importance on projection (VIP) and false discovery rate (FDR) estimation techniques, we were able to conduct a better assessment of a great number of responses, and this enabled us to estimate with a high level of confidence, the significant and discriminant chemical variables for the differentiation and correlation of the seasons. The results of this study showed that the concentration of different compounds was directly affected by the harvest period. The differences observed between the samples may be related to the influence of the environmental conditions that are typically characteristic of each period, such as temperature, solar radiation, relative humidity, and rainfall.

*B. intermedia* and *S. marginata* are plant species that belong to the Brazilian savanna, known as Brazil’s Cerrado biome, and for this reason, the harvest location of these species presented very similar weather conditions. The climatological characteristics of the Cerrado indicate the existence of two main periods/seasons: the long, dry and warm season [April/May (autumn) to August/September (winter)] and the short, rainy and hot season [September/October (spring) to February/March (summer)]. According to some studies reported in the literature, drought is the main environmental stress factor that affects plant yield and quality; this factor particularly affects the accumulation of bioactive compounds in the plants, leading to significant changes in the amount of phenolic compounds present in the chemical composition of these plants (Wang et al., 2016; Almeida et al., 2020; Lambers et al., 2020). Water deficiency in periods of drought enhances the formation of reactive oxygen species (ROS), and this leads the plant to activate its defense mechanism by changing the biosynthesis pathway in response to drought stress to produce antioxidant compounds such as, flavonol glycosides and galloylquinic acid derivatives for *B. intermedia* samples and *C*-glycosyl flavones for *S. marginata*, which have the ability to capture free radicals and reduce the oxidative damage (Paudel et al., 2016). Moreover, after a long period of drought stress, both *B. intermedia* and *S. marginata* experience a reduction in the content of phenolic compounds; this is particularly evident in the spring period (Ahmed et al., 2019).

Interestingly, a study carried out by Velasque and Del-Claro (2016), reported slightly different findings; according to this study, the production of new leaves in the species *B. intermedia* follows a seasonal pattern and occurs after rainy periods with predominant growth in dry periods. Thus, the increased levels of phenolic compounds during autumn and winter may also be associated with the defense mechanism of the plant which is related to the resistance of young leaf tissues to plant pathogens (Andrade et al., 2017).

The extremely high levels of triterpenes present in *B. intermedia* during the summer indicates that the accumulation of these compounds strongly depends on warm weather and higher light intensity, which are the predominant environmental factors during this season (Alqahtani et al., 2015). Higher temperatures can lead to higher evaporation rates and decreased water availability, and these factors cause negative effects on the development and growth of the plant (Paudel et al., 2016; de Araújo Silva et al., 2021). In this way, plants avoid dehydration by producing a lipophilic layer coating on the surface of the leaves (Buschhaus and Jetter, 2012). Studies in the literature, have shown that triterpenes are the major components of this cuticular layer present in many plants (Buschhaus and Jetter, 2011; Moses et al., 2014). Furthermore, this barrier is a plant-environment interface against abiotic stresses, and its functions include blocking the loss of water, deterring insects and pathogens, obstructing the penetration of UV rays, protecting the plants from high temperatures and other potential threats that the plant may be exposed to (González-Coloma et al., 2011; Buschhaus and Jetter, 2012; Diarte et al., 2021).

The different concentrations of saponins observed in different harvest periods of *S. marginata* showed that the plant recorded higher levels of saponins during the spring and autumn but had extremely low levels of saponins during the winter. Abiotic stress factors, such as humidity, high temperatures and solar radiation may have influenced the content of saponins in *S. marginata* during the autumn/spring seasons, while drought conditions during the winter may have contributed to the decrease in the content of saponins in the plant. In line with these observations, studies published in the literature have shown that, for some plant species, that environmental stresses associated with increased saponin levels, are responsible for the mechanism of activation of some signaling agents that promote the biosynthesis of these compounds (Szakiel et al., 2011; de Costa et al., 2013; Moses et al., 2014).

It is worth noting that, in general, saponins possess antimicrobial, antifungal, antiparasitic and insecticidal properties, and these properties enable them to play a key role in the plant defense mechanism against pathogenic microbes, pests and herbivores (Sparg et al., 2004; Moses et al., 2014; Biswas and Dwivedi, 2019).

Other studies, reported that the presence of higher amounts of hederagenin-based saponins in some plants is associated with the plant defense response against herbivores; according to these authors the hydroxylation that occurs at the C-23 position of oleanolic acid and which promotes the formation of the hederagenin structure, is a modification in the chemical composition of the plant which enhances its defense mechanism (Liu et al., 2019).

However, the relationship between metabolite variation and the environmental factors need to be carefully analyzed, since there may be a gap in the physiological temporal response of the plant (Castro et al., 2017). In addition, bushfire (both natural and human) (Lambers et al., 2020), low soil nutrients (Lambers et al., 2020) and high rates of evapotranspiration (Almeida et al., 2020) are common factors that occur in the Cerrado (savanna) region; all these factors could affect the accumulation of compounds in *B. intermedia* and *S. marginata*.

This study brings novel findings to light regarding *B. intermedia* and *S. marginata* and provides relevant insights into the identification of specialized metabolites related to environmental conditions which may be useful for establishing standardization criteria for plant extracts aiming at improving the quality control of medicinal plants.

The results obtained from the present study also showed the relevant role played by the secondary metabolites in the defense mechanisms against stresses caused by environmental factors in each plant species. Drought, UV radiation and temperature were the main stress factors that were found to induce the formation of ROS and the accumulation of specialized metabolites with antioxidant capacity in the plants. The strong antioxidant activity of many of these metabolites was also found to have beneficial effects on human health. The findings of this study showed that the plant species investigated here have suitable medicinal properties which can be useful for the treatment of inflammatory diseases.

## Data Availability

The raw data supporting the conclusion of this article will be made available by the authors, without undue reservation.
