# Corrigendum: UHPLC-(ESI)-HRMS and NMR-Based Metabolomics Approach to Access the Seasonality of *Byrsonima intermedia* and *Serjania marginata* From Brazilian Cerrado Flora Diversity

**DOI:** 10.3389/fchem.2021.737969

**Published:** 2021-10-29

**Authors:** Ana C. Zanatta, Wagner Vilegas, RuAngelie Edrada-Ebel

**Affiliations:** ^1^ Laboratory of Phytochemistry, Institute of Chemistry, Department of Biochemistry and Organic Chemistry, São Paulo State University (UNESP), Araraquara, Brazil; ^2^ Laboratory of Bioprospecting of Natural Products, Institute of Biosciences, São Paulo State University (UNESP), São Vicente, Brazil; ^3^ Strathclyde Institute of Pharmacy and Biomedical Sciences, University of Strathclyde, Glasgow, United Kingdom

**Keywords:** metabolomic approach, specialized metabolites, seasonality, environmental factors, phenolic compounds, saponins, triterpenes

In the original article, there was an error. The words “and *T. catappa*” should be removed.

A correction has been made to **Materials and Methods Section**, under subsection *Data processing*, Paragraph Number: 3:

“The ^1^H NMR and *J*-res projection spectra were previously stacked and then pre-processing according the following steps: baseline correction with the Whittaker Smoother option; apodization with Gaussian function of 1 GB [Hz]; normalization by the largest peak and smoothing Savitzky-Golay method. The processed NMR data were prepared using bin width of 0.04 ppm and the bin intensities method was average sum. Afterwards, the spectral data were saved with the peak intensities. Chemical shifts (*δ*) values were established between 0.0 and 10.0 ppm for *S. marginata* samples data and 0.0–13.0 ppm for *B. intermedia*.”

In the original article, there was an error. The words “and *T. catappa*” should be removed.

A correction has been made to **Discussion Section**, Paragraph Number 1:

“The analyses conducted for each set of samples using LC-HRMS and NMR showed that the extracts prepared from the leaves of the plant species *B. intermedia* and *S. marginata* had a comparable metabolite profile.”

In the original article, there weres errors. The words “and 90 compounds in the extracts of *T. catappa*” should be removed, and the reference to “Supplementary Table S7” should be “Supplementary Table S5.”

A correction has been made to **Discussion Section**, Paragraph Number 3:

“The dereplication of the ESI-HRMS data using the macro database containing the Natural Products Dictionary (DNP) allowed a rapid and effective annotation of 68 compounds in the extracts of *B. intermedia* and 81 compounds in the extracts of *S. marginata* (see **Supplementary Tables S4, S5**).”

In the original article, there was an error. The reference to “Supplementary Table S5” should be “Supplementary Table S4.”

A correction has been made to **Discussion Section**, Paragraph Number 4:

“As mentioned previously in the results section, the metabolite profile of the hydroethanolic extracts of *B. intermedia* revealed that this plant is a rich source of cinnamic acids, phenolic acids derived from galloyl quinic and shikimic acid, proanthocyanidins, glycosylated flavonoids, triterpenes and other phenols (**Supplementary Table S4**).”

In the original article, there was an error. A reference to “Supplementary Table S6” should be “Supplementary Table S5.”

A correction has been made to **Discussion Section**, Paragraph Number 8:

“The extracts of *S. marginata* showed mainly exhibited compounds derived from saponins, glycosylated flavonoids, catechins and oligomeric proanthocyanidins (**Supplementary Table S5**). The detection of saponins in *S. marginata* in this study helped to confirm the findings reported in the literature which pointed out that saponins are the major class of compounds in this plant species (**Heredia-Vieira et al., 2015**). Precisely, the results of our study showed that triterpenic saponins were prevalent in the extracts of *S. marginata*; these compounds contained aglycones of oleanolic acid (olean-12-en-28-oic), hederagenin (3,23-dihydroxyolean-12-en-28-acid) and gypsogenin (3 hydroxy-23-oxo-olean-12-en-28-acid). The presence of glycosylated flavonoids was detected in three groups: *C*-glycosylated, *C,O*-glycosylated and *O*-glycosylated flavonoids (**Supplementary Table S5**). The compounds tetrastigma B (*m/z* 561.1603 [M+H]^+^, R_t_ = 9.89) and cassiaocidentalin A (*m/z* 561.1602 [M+H]^+^, R_t_ = 10.37) are examples of *C,O* glycosylated flavonoids isomers with an interglycosidic linkage between a deoxyhexose and 6-dideoxyhexose sugars (**Heredia-Vieira et al., 2015**). Two types of proanthocyanidin compounds were found in *S. marginata*; these included B-type and A-type proanthocyanidins (**Supplementary Table S5**). B-type proanthocyanidins are characterized by a single interflavan linkage between monomeric units which can be formed by the equivalent units of (epi)catechin. A-type proanthocyanidins, on the other hand, contain double interflavan bonds.”

The authors apologize for these errors and state that this does not change the scientific conclusions of the article in any way. The original article has been updated.

