# Supplementary material for: UHPLC-(ESI)-HRMS and NMR-Based Metabolomics Approach to Access the Seasonality of Byrsonima intermedia and Serjania marginata From Brazilian Cerrado Flora Diversity
Source: Front Chem. 2021 Jul 6;9:710025. doi: 10.3389/fchem.2021.710025 (PMC8290060; doi:10.3389/fchem.2021.710025)
Supplement: Supplementary file 1 [file DataSheet1.DOCX]

Supplementary Material

# Supplementary Figures and Tables

## Supplementary Figures

**B**

**A**

| 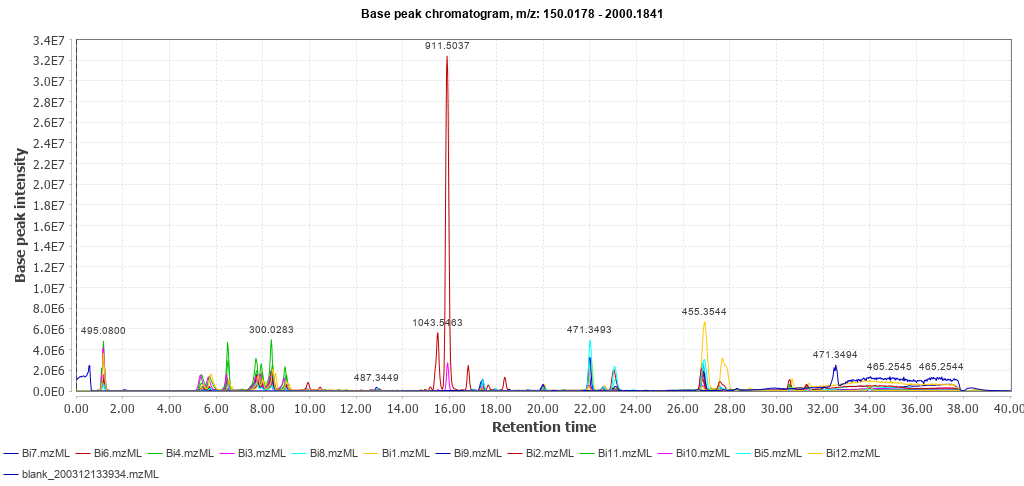  **A** |
| --- |
| 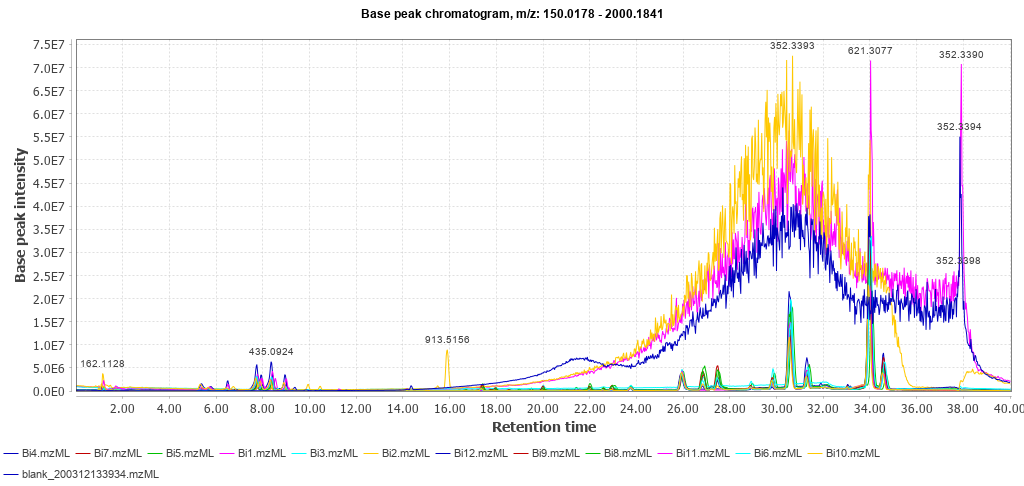 |

**Supplementary Figure 1.** Base peak chromatogram for *B. intermedia* samples obtained from pre-processing by MZmine2. **(A)** negative ionization mode; **(B)** positve ionization mode.

**B**

| 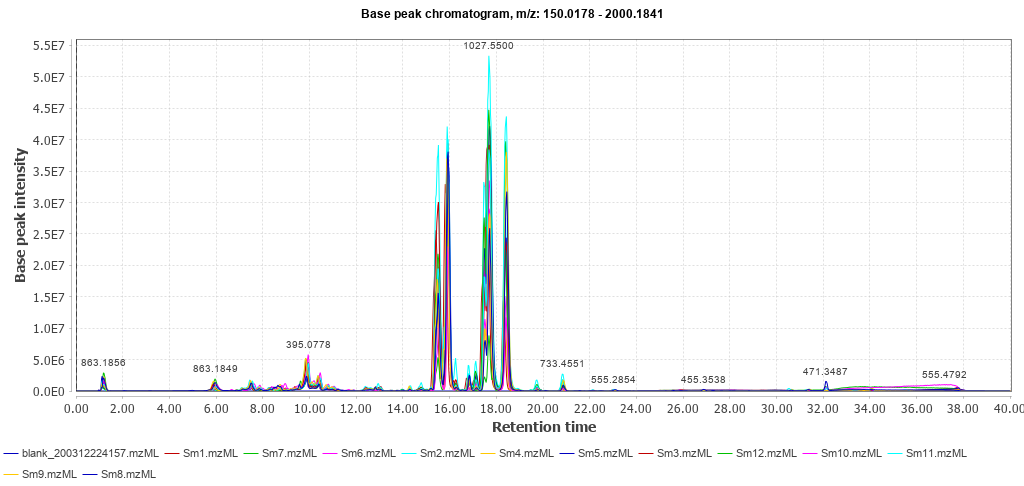  **B**  **A** |
| --- |
| 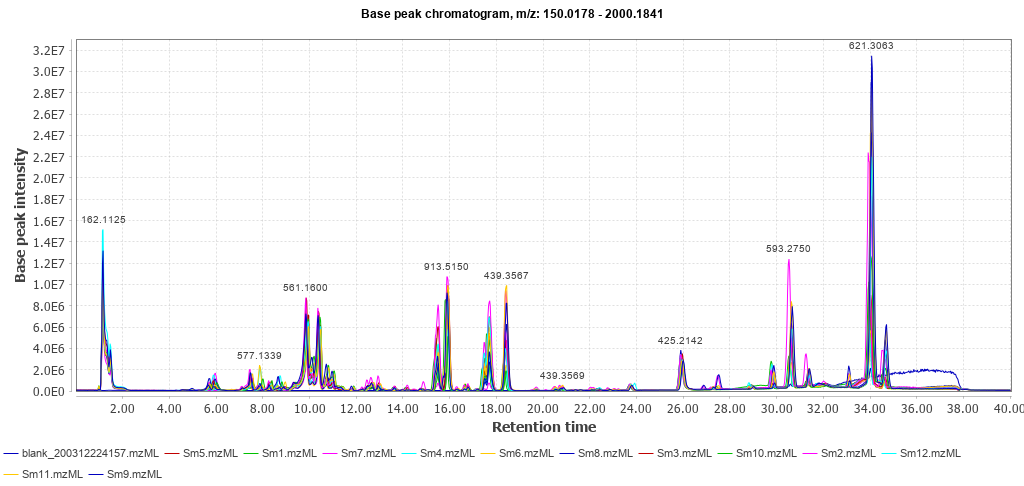 |

Supplementary Figure 2. Base peak chromatogram for *S. marginata* samples obtained from pre-processing by MZmine2. (A) negative ionization mode; (B) positve ionization mode.

**B**

**B**

Supplementary Figure 3. ^1^H NMR spectra (DMSO-d_6_, 500 MHz) of *B. intermedia* samples (Bi1 to Bi12).

Supplementary Figure 4. ^1^H NMR spectra (DMSO-d_6_, 500 MHz) of *S. marginata* samples (Sm1 to Sm12).

Supplementary Figure 5. One-dimensional *J*-resolved NMR spectra (DMSO-d_6_, 500 MHz) of *B. intermedia* samples (Bi1 to Bi12).

Supplementary Figure 6. 2D *J*-resolved NMR spectra (DMSO-d_6_, 500 MHz) of *B. intermedia* samples (Bi1 to Bi12).

Supplementary Figure 7. One-dimensional *J*-resolved NMR spectra (DMSO-d_6_, 500 MHz) of *S. marginata* samples (Sm1 to Sm12).

Supplementary Figure 8. 2D *J*-resolved NMR spectra (DMSO-d_6_, 500 MHz) of *S. marginata* samples (Sm1 to Sm12).


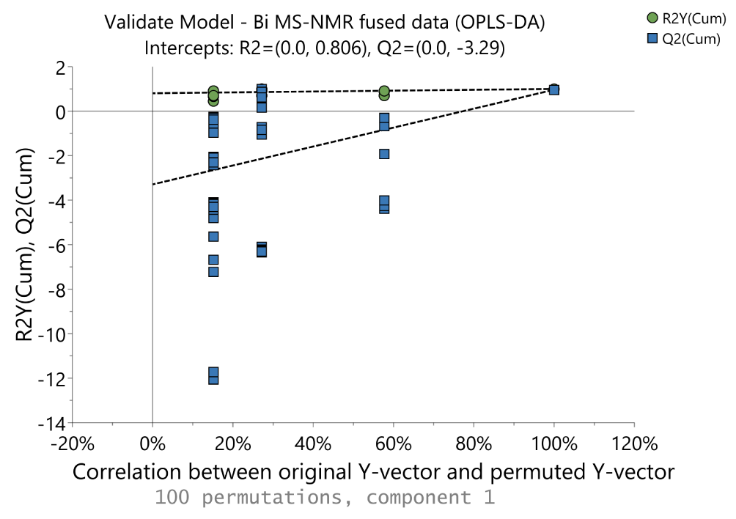

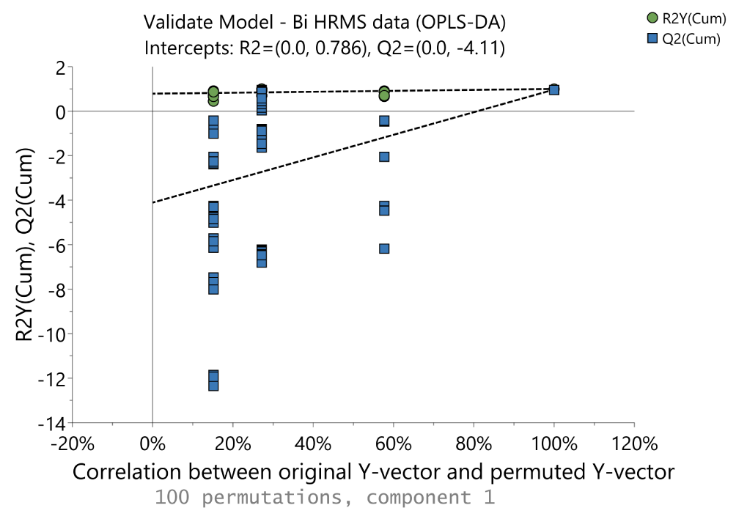


**C**

**D**

**B**

**A**


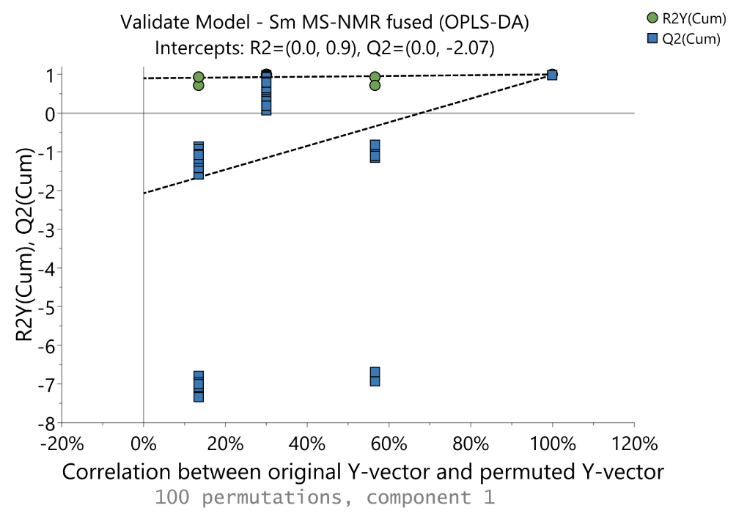

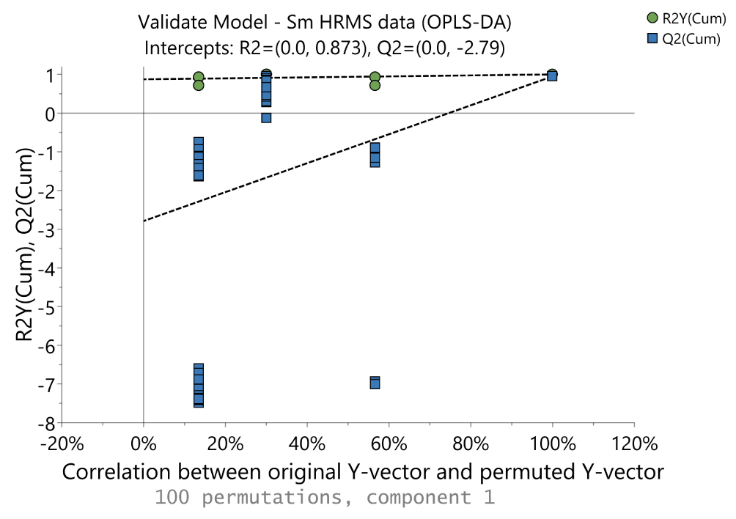


Supplementary Figure 9. Permutation plot of OPLS-DA validated model for (A) *B. intermedia* MS-NMR fused data; (B) *B. intermedia* HRMS data; (C) *S. marginata* MS-NMR fused data, and (D) *S. marginata* HRMS data.


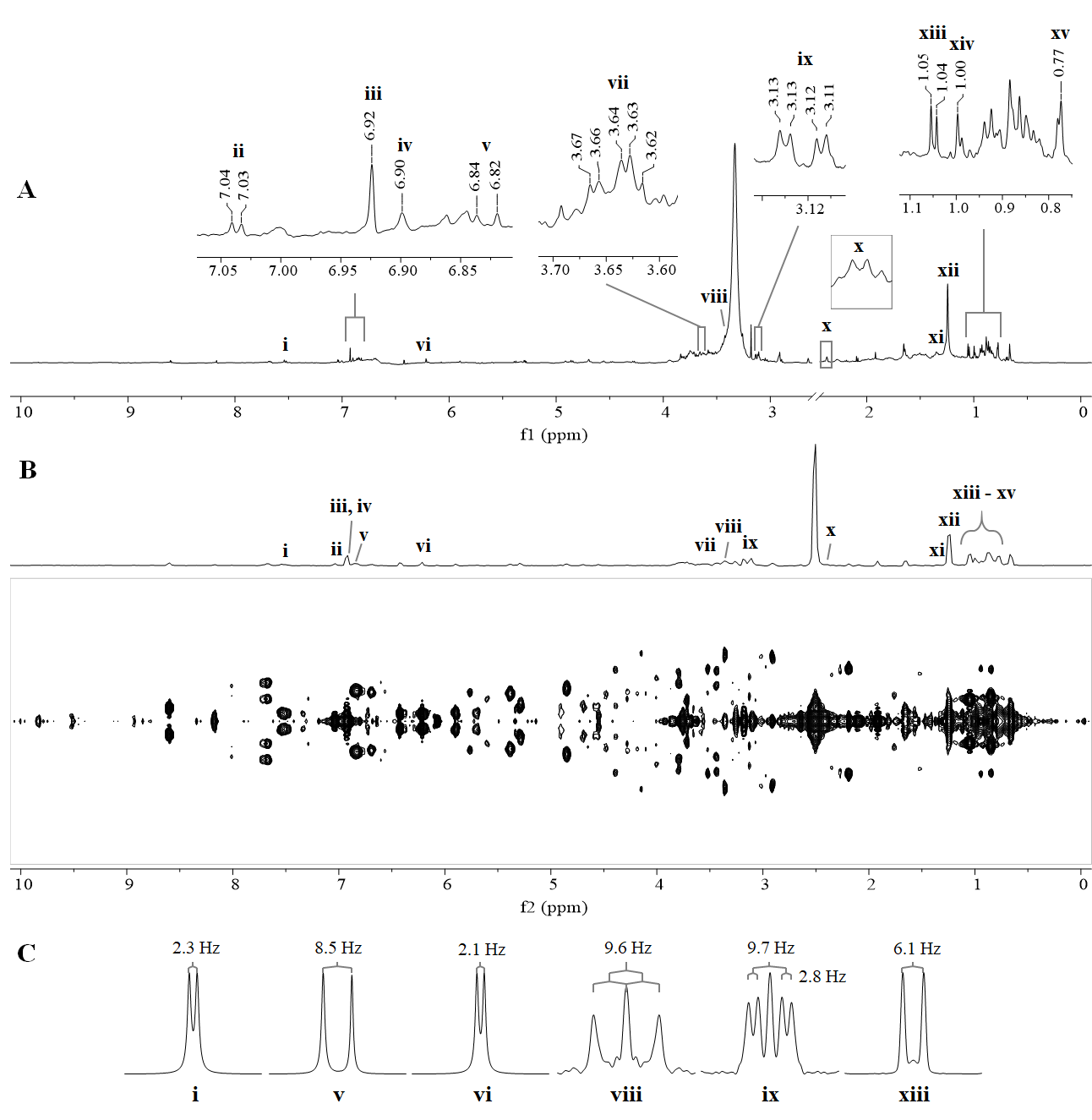


Supplementary Figure 10. (A) Representative ^1^H NMR spectrum (DMSO-d_6_, 500 MHz) of *B. intermedia* extracts. (B) Representative 2D *J*-res NMR spectrum (DMSO-d_6_, 500 MHz) of *B. intermedia* extracts. (C) Traces for the highlighted peaks regions through the F1′ dimension with related *J* coupling constants. Signals peaks i-xv are the top discriminant 15 variables according to the VIP scores: (i) *δ* 7.53 (137), (ii) *δ* 7.04 (150), (iii) *δ* 6.93 (152), (iv) *δ* 6.90 (153), (v) *δ* 6.83 (155), (vi) *δ* 6.21 (170), (vii) *δ* 3.62 (235), (viii) *δ* 3.37 (241), (ix) *δ* 3.12 (248), (x) *δ* 2.39 (266), (xi) *δ* 1.35 (292), (xii) *δ* 1.24 (295), (xiii) *δ* 1.04 (300), (xiv) *δ* 1.00 (301) and (xv) *δ* 0.77 (307).


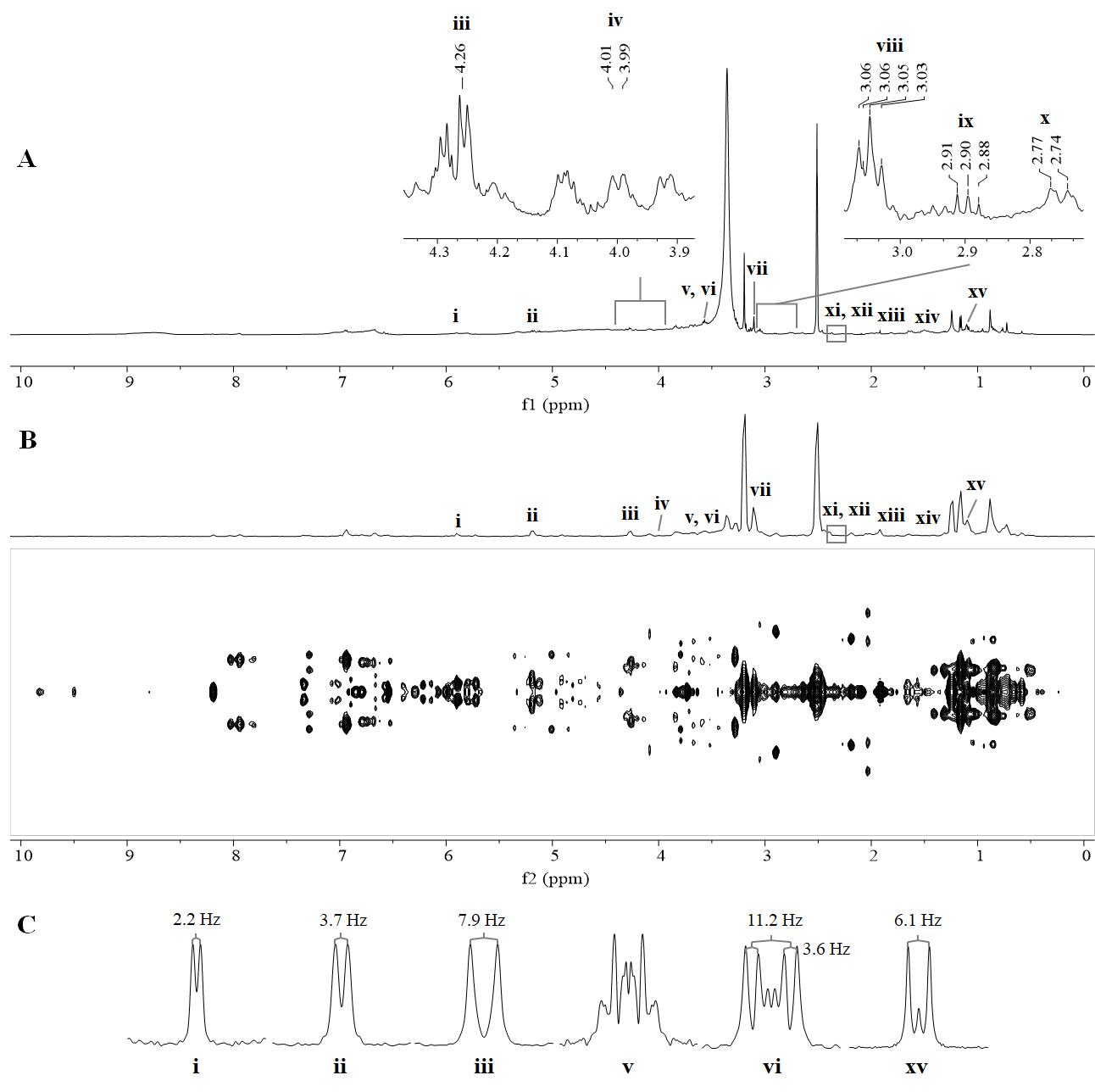


Supplementary Figure 11. (A) Representative ^1^H NMR spectrum (DMSO-d_6_, 500 MHz) of *S. marginata* extracts. (B) Representative 2D *J*-res NMR spectrum (DMSO-d_6_, 500 MHz) of *S. marginata* extracts. (C) Traces for the highlighted peaks regions through the F1′ dimension with related *J* coupling constants. Signals peaks i-xv are the top discriminant 15 variables according to the VIP scores: (i) *δ* 5.90 (103), (ii) *δ* 5.19 (121), (iii) *δ* 4.26 (144), (iv) *δ* 3.99 (151), (v) *δ* 3.58 (161), (vi) *δ* 3.52 (163), (vii) *δ* 3.18 (171), (viii) *δ* 3.03 (175), (ix) *δ* 2.91 (178), (x) *δ* 2.72 (182), (xi) *δ* 2.39 (191), (xii) *δ* 2.27 (194), (xiii) *δ* 1.82 (205), (xiv) *δ* 1.49 (213) and (xv) *δ* 1.08 (224).

|   **A** |
| --- |
|  |

**Supplementary Figure 12. Relative distribution of discriminant metabolites in *B. intermedia* extracts.** **(A)** Box-plots representing the concentration values of discriminant metabolites signals with FDR ≤ 0.05 (*δ* in ppm - ID) using MS-NMR fused data. **(B)** Box-plots representing the concentration values of discriminant metabolites signals with FDR ≤ 0.05 using HRMS data (*m/z* - MZmine ID).

**B**

**A**

|   **A** |
| --- |
|  |

**Supplementary Figure 13. Relative distribution of discriminant metabolites in *S. marginata* extracts.** **(A)** Box-plots representing the concentration values of discriminant metabolites signals with FDR ≤ 0.05 (*δ* in ppm - ID) using MS-NMR fused data. **(B)** Box-plots representing the concentration values of discriminant metabolites signals with FDR ≤ 0.05 using HRMS data (*m/z* - MZmine ID).

**B**

**A**

## Supplementary Tables

Supplementary Table 1. Harvesting period of leaves of the plant species *B. intermedia* and *S. marginata*.

| ***B. intermedia*** | | | ***S. marginata*** | | |
| --- | --- | --- | --- | --- | --- |
| **Code** | **Month** | **Season** | **Code** | **Month** | **Season** |
| Bi1 | Mar/17 | Summer | Sm1 | Feb/17 | Summer |
| Bi2 | Apr/17 | Autumn | Sm2 | Apr/17 | Autumn |
| Bi3 | Jun/17 | Autumn | Sm3 | Jun/17 | Autumn |
| Bi4 | Aug/17 | Winter | Sm4 | Aug/17 | Winter |
| Bi5 | Oct/17 | Spring | Sm5 | Oct/17 | Spring |
| Bi6 | Dec/17 | Spring | Sm6 | Dec/17 | Summer |
| Bi7 | Feb/18 | Summer | Sm7 | Feb/18 | Autumn |
| Bi8 | Apr/18 | Autumn | Sm8 | Jun/18 | Autumn |
| Bi9 | Jun/18 | Autumn | Sm9 | Jul/18 | Winter |
| Bi10 | Aug/18 | Winter | Sm10 | Aug/18 | Winter |
| Bi11 | Oct/18 | Spring | Sm11 | Nov/18 | Spring |
| Bi12 | Dec/18 | Spring | Sm12 | Dec/18 | Summer |

Supplementary Table 2. Weather characteristics for Bauru-SP in 2017 and 2018: average temperature, solar radiation, air humidity and rainfall.

| **Period** | **Average of temperature (°C) per month** | **Average of solar radiation (kJ/m^2^) per month** | **Average relative air humidity (%)** | **Cumulative amount of rainfall (mm) per month** |
| --- | --- | --- | --- | --- |
| Mar/17 | 26.9 | 2328.87 | 68 | 0.17 |
| Apr/17 | 21.7 | 1755.32 | 79 | 0.14 |
| Jun/17 | 16.0 | 325.76 | 92 | 0.03 |
| Aug/17 | 14.2 | 401.02 | 88 | 0.08 |
| Oct/17 | 20.1 | 2639.78 | 82 | 0.14 |
| Dec/17 | 25.4 | 1818.86 | 72 | 0.13 |
| Feb/18 | 24.6 | 2012.24 | 69 | 0.12 |
| Apr/18 | 19.1 | 1912.28 | 84 | 0.02 |
| Jun/18 | 15.3 | 495.75 | 81 | 0.02 |
| Aug/18 | 17.6 | 740.31 | 69 | 0.08 |
| Oct/18 | 19.6 | 2216.41 | 97 | 0.22 |
| Dec/18 | 28.9 | 2782.35 | 54 | 0.22 |

The climatic data were provided by the Meteorological Data Storage Section (SADMET) of the National Institute of Meteorology (INMET). Accessed at: [www.inmet.gov.br](http://www.inmet.gov.br/).

Supplementary Table 3. Weather characteristics for Dourados-MS in 2017 and 2018: average temperature, solar radiation, air humidity and rainfall.

| **Period** | **Average of temperature (°C) per month** | **Average of solar radiation (kJ/m^2^) per month** | **Average relative air humidity (%)** | **Cumulative amount of rainfall (mm) per month** |
| --- | --- | --- | --- | --- |
| Feb/17 | 19.6 | 18.44 | 93 | 0.07 |
| Apr/17 | 20.7 | 29.13 | 74 | 0.05 |
| Jun/17 | 15.8 | 0.42 | 96 | 0.00 |
| Aug/17 | 13.6 | 1.88 | 97 | 0.07 |
| Oct/17 | 14.6 | 158.22 | 91 | 0.13 |
| Dec/17 | 21.2 | 72.54 | 95 | 0.00 |
| Feb/18 | 23.6 | 8.73 | 91 | 0.18 |
| Jun/18 | 22.2 | 0.45 | 68 | 0.13 |
| Jul/18 | 10.3 | 0.18 | 86 | 0.00 |
| Aug/18 | 19.6 | 0.97 | 77 | 0.09 |
| Nov/18 | 20.9 | 183.96 | 82 | 0.26 |
| Dec/18 | 22.4 | 186.81 | 97 | 0.12 |

The climatic data were provided by the Meteorological Data Storage Section (SADMET) of the National Institute of Meteorology (INMET). Accessed at: [www.inmet.gov.br](http://www.inmet.gov.br/).

Supplementary Table 4. Proposed compounds for seasonal harvests of *B. intermedia* extracts of by UHPLC-(ESI)-HRMS.

| **No.** | **Rt (min)** | **Adduct (*m/z*)** | **MW** | **Chemical formula** | **Error (ppm)** | **Annotation of metabolite class or compound** |
| --- | --- | --- | --- | --- | --- | --- |
| **1** | 1.55 | 333.0821  [M+H]^+^ | 332.0748 | C_13_H_16_O_10_ | 1.4 | *O*-Galloylhexose |
| **2** | 1.73 | 169.0137  [M-H]^-^ | 170.0210 | C_7_H_6_O_5_ | -0.1 | Gallic acid |
| **3** | 2.01 | 191.0556  [M-H]^-^ | 192.0629 | C_7_H_12_O_6_ | 0.1 | Quinic acid |
| **4** | 2.25 | 479.0825  [M+H]^+^ | 478.0752 | C_21_H_18_O_13_ | 1.0 | Di-*O*-galloylshikimic acid |
| **5** | 2.36 | 497.0930  [M+H]^+^ | 496.0857 | C_21_H_20_O_14_ | 0.9 | Di-*O*-galloylquinic acid |
| **6** | 3.22 | 479.0824  [M+H]^+^ | 478.0752 | C_21_H_18_O_13_ | 0.9 | Di-*O*-galloylshikimic acid |
| **7** | 3.35 | 649.1040  [M+H]^+^ | 648.0968 | [C_28_H_24_O_18_](https://pubchem.ncbi.nlm.nih.gov/#query=C28H24O18) | 0.8 | Tri-*O*-galloylquinic acid |
| **8** | 3.60 | 497.0930  [M+H]^+^ | 496.0857 | C_21_H_20_O_14_ | 0.8 | Di-*O*-galloylquinic acid |
| **9** | 4.31 | 291.0864  [M+H]^+^ | 290.0791 | C_15_H_14_O_6_ | 0.3 | (epi)catechin |
| **10** | 4.89 | 597.1456  [M+H]^+^ | 596.1383 | C_26_H_28_O_16_ | 1.0 | Quercetin-*O*-hexose-pentose |

Supplementary Table 4. *(continued)*

| **No.** | **Rt (min)** | **Adduct (*m/z*)** | **MW** | **Chemical formula** | **Error (ppm)** | **Annotation of metabolite class or compound** |
| --- | --- | --- | --- | --- | --- | --- |
| **11** | 5.40 | 479.0826  [M+H]^+^ | 478.0753 | C_21_H_18_O_13_ | 1.2 | Di-*O*-galloylshikimic acid |
| **12** | 5.40 | 649.1042  [M+H]^+^ | 648.0970 | [C_28_H_24_O_18_](https://pubchem.ncbi.nlm.nih.gov/#query=C28H24O18) | 1.1 | Tri-*O*-galloylquinic acid |
| **13** | 5.41 | 497.0932  [M+H]^+^ | 496.0859 | C_21_H_20_O_14_ | 1.1 | Di-*O*-galloylquinic acid |
| **14** | 5.57 | 327.0713  [M+H]^+^ | 326.0641 | C_14_H_14_O_9_ | 0.9 | *O*-Galloylshikimic acid |
| **15** | 5.58 | 345.0819  [M+H]^+^ | 344.0747 | C_14_H_16_O_10_ | 0.9 | *O*-Galloylquinic acid |
| **16** | 5.72 | 291.0865  [M+H]^+^ | 290.0792 | C_15_H_14_O_6_ | 0.5 | (epi)catechin |
| **17** | 5.72 | 631.0937  [M+H]^+^ | 630.0865 | C_28_H_22_O_7_ | 1.2 | Tri-*O*-galloylshikimic acid |
| **18** | 5.74 | 801.1151  [M+H]^+^ | 800.1079 | C_33_H_41_O_20_ | 0.8 | Tetra-*O*-galloylquinic acid |
| **19** | 5.79 | 339.1078  [M+H]^+^ | 338.1005 | C_16_H_18_O_8_ | 1.0 | Coumaroylquinic acid |
| **20** | 5.85 | 799.1035  [M-H]^-^ | 800.1107 | C_33_H_41_O_20_ | 4.3 | Tetra-*O*-galloylquinic acid |
| **21** | 6.18 | 1153.2609  [M+H]^+^ | 1152.2537 | C_60_H_48_O_24_ | 0.1 | (epi)catechin-(epi)catechin-A-(epi)catechin-(epi)catechin |
| **22** | 6.39 | 355.1025  [M+H]^+^ | 354.0952 | C_16_H_18_O_9_ | 0.3 | Caffeoylquinic acid (chlorogenic acid) |
| **23** | 6.52 | 757.2195  [M+H]^+^ | 756.2123 | C_33_H_40_O_20_ | 1.3 | Quercetin-*O*-hexose-deoyhexose-deoxyhexose |
| **24** | 6.57 | 631.0939  [M+H]^+^ | 630.0866 | C_28_H_22_O_7_ | 1.4 | Tri-*O*-galloylshikimic acid |
| **25** | 6.82 | 199.0605  [M+H]^+^ | 198.0532 | C_9_H_10_O_5_ | 1.8 | Syringic acid |
| **26** | 6.91 | 369.1183  [M+H]^+^ | 368.1111 | C_17_H_20_O_9_ | 0.9 | Feruloylquinic acid |
| **27** | 7.09 | 213.0761  [M+H]^+^ | 212.0689 | C_10_H_12_O_5_ | 1.8 | Trimethyl gallate |
| **28** | 7.16 | 617.1146  [M+H]^+^ | 616.1073 | C_28_H_24_O_16_ | 1.4 | Quercetin-*O*-(*O*-galloyl)-hexose |
| **29** | 7.51 | 611.1617  [M+H]^+^ | 610.1542 | C_27_H_30_O_16_ | 1.6 | Quercetin-*O*-hexose-deoxyhexose |
| **30** | 7.65 | 881.1602  [M-H]^-^ | 882.1675 | [C_44_H_34_O](https://pubchem.ncbi.nlm.nih.gov/#query=C28H24O18)_20_ | 3.4 | *O*-Galloyl-(epi)catechin-(epi)catechin-*O* gallate |
| **31** | 7.68 | 443.0977  [M+H]^+^ | 442.0904 | C_22_H_18_O_10_ | 0.9 | (epi)catechin gallate |
| **32** | 7.78 | 465.1035  [M+H]^+^ | 464.0963 | C_21_H_20_O_12_ | 1.7 | Quercetin-*O*-hexose |
| **33** | 7.83 | 577.1351  [M+H]^+^ | 576.1279 | C_30_H_24_O_12_ | 1.9 | (epi)catechin-A-(epi)catechin |

Supplementary Table 4. *(continued)*

| **No.** | **Rt (min)** | **Adduct (*m/z*)** | **MW** | **Chemical formula** | **Error (ppm)** | **Annotation of metabolite class or compound** |
| --- | --- | --- | --- | --- | --- | --- |
| **34** | 7.83 | 163.0396  [M-H]^-^ | 164.0469 | C_9_H_8_O_3_ | -2.6 | Coumaric acid |
| **35** | 7.97 | 617.1147  [M+H]^+^ | 616.1074 | C_28_H_24_O_16_ | 1.5 | Quercetin-*O*-(*O*-galloyl)-hexose |
| **36** | 8.03 | 581.1511  [M+H]^+^ | 580.1439 | C_26_H_28_O_15_ | 1.8 | Quercetin-*O*-deoxyhexose-pentose |
| **37** | 8.38 | 183.0298  [M-H]^-^ | 184.0371 | C_8_H_8_O_5_ | -0.6 | Methyl gallate |
| **38** | 8.40 | 435.0928  [M+H]^+^ | 434.0855 | C_20_H_18_O_11_ | 1.4 | Quercetin-*O*-pentose |
| **39** | 8.44 | 449.1085  [M+H]^+^ | 448.1012 | C_21_H_20_O_11_ | 1.5 | Quercetin-*O*-deoxyhexose |
| **40** | 8.53 | 497.0933  [M+H]^+^ | 496.0861 | C_21_H_20_O_14_ | 1.5 | Di-*O*-galloylquinic acid |
| **41** | 8.75 | 601.1197  [M+H]^+^ | 600.1124 | C_28_H_24_O_15_ | 1.5 | Quercetin-*O*-(*O*-galloyl)-deoxyhexose |
| **42** | 8.91 | 173.0606  [M-H]^-^ | 174.0679 | C_7_H_10_O_5_ | -1.0 | Shikimic acid |
| **43** | 8.95 | 577.1353  [M+H]^+^ | 576.1280 | C_30_H_24_O_12_ | 2.2 | (epi)catechin-A-(epi)catechin |
| **44** | 8.96 | 631.1309  [M+H]^+^ | 630.1236 | C_29_H_26_O_16_ | 2.4 | *O*-Coumaroyl-di-*O*-galloyl-hexose |
| **45** | 8.99 | 587.1041  [M+H]^+^ | 586.0969 | C_27_H_22_O_15_ | 1.7 | Quercetin-*O*-(*O*-galloyl)-pentose |
| **46** | 9.52 | 275.0917  [M+H]^+^ | 274.0845 | C_15_H_14_O_5_ | 1.2 | (epi)afzelechin |
| **47** | 9.66 | 933.2285  [M-H]^-^ | 934.2357 | C_42_H_46_O_24_ | -2.3 | Quercetin-*O*-(*O*-coumaroyl)-hexose-hexose-hexose |
| **48** | 11.02 | 487.1243  [M+H]^+^ | 486.1170 | C_24_H_22_O_11_ | 1.6 | Dimethyl (epi)gallocatechin gallate |
| **49** | 12.02 | 531.1503  [M+H]^+^ | 530.1430 | C_26_H_26_O_12_ | 1.1 | Di-*O*-caffeoylquinic acid methyl ester |
| **50** | 12.55 | 531.1505  [M+H]^+^ | 530.1432 | C_26_H_26_O_12_ | 1.5 | Di-*O*-caffeoylquinic acid methyl ester |
| **51** | 13.87 | 333.1336  [M+H]^+^ | 332.1263 | C_18_H_20_O_6_ | 1.0 | (epi)catechin trimethyl ether |
| **52** | 14.19 | 289.2376  [M+H]^+^ | 288.2303 | C_16_H_32_O_4_ | 0.7 | Fatty acid |
| **53** | 17.92 | 489.3579  [M+H]^+^ | 488.3506 | C_30_H_48_O_5_ | 1.0 | Trihydroxy-12-oleanen-28-oic acid |
| **54** | 19.62 | 487.3422  [M+H]^+^ | 486.3349 | C_30_H_46_O_5_ | 0.8 | Trihydroxy-5,12-oleanadien-28-oic acid |
| **55** | 20.05 | 489.3580  [M+H]^+^ | 488.3507 | C_30_H_48_O_5_ | 1.1 | Trihydroxy-12-ursen-28-oic acid |

Supplementary Table 4. *(continued)*

| **No.** | **Rt (min)** | **Adduct (*m/z*)** | **MW** | **Chemical formula** | **Error (ppm)** | **Annotation of metabolite class or compound** |
| --- | --- | --- | --- | --- | --- | --- |
| **56** | 26.88 | 455.3546  [M-H]^-^ | 456.3619 | C_30_H_48_O_3_ | 3.4 | Betulinic acid |
| **57** | 26.91 | 439.3576  [M+H]^+^ | 438.3503 | C_30_H_46_O_2_ | 1.3 | Betulonic aldehyde |
| **58** | 27.61 | 455.3547  [M-H]^-^ | 456.3619 | [C_30_H_48_O_3_](https://pubchem.ncbi.nlm.nih.gov/#query=C30H48O3) | 3.4 | Oleanolic acid |
| **59** | 27.65 | 439.3576  [M+H]^+^ | 438.3503 | C_30_H_46_O_2_ | 1.2 | 3-oxo-olean-12-en-28-al |
| **60** | 28.08 | 607.2560  [M+H]^+^ | 606.2487 | C_35_H_34_N_4_O_6_ | 1.4 | Phaeophorbide B |
| **61** | 30.34 | 621.2719  [M+H]^+^ | 620.2647 | C_36_H_36_N_4_O_6_ | 1.9 | Methyl phaeophorbide B |
| **62** | 30.62 | 593.2767  [M+H]^+^ | 592.2694 | C_35_H_36_N_4_O_5_ | 1.5 | Phaeophorbide A |
| **63** | 31.34 | 635.2876  [M+H]^+^ | 634.2803 | C_37_H_38_N_4_O_6_ | 1.8 | Ethyl phaeophorbide B |
| **64** | 33.18 | 607.2926  [M+H]^+^ | 606.2853 | C_36_H_38_N_4_O_5_ | 1.8 | Methyl phaeophorbide A |
| **65** | 34.00 | 643.2902  [M+H]^+^ | 642.2829 | C_38_H_42_O_9_ | < 0.1 | Diterpene |
| **66** | 34.00 | 621.3085  [M+H]^+^ | 620.3012 | C_37_H_40_N_4_O_5_ | 2.1 | Ethyl phaeophorbide A |
| **67** | 34.59 | 621.3085  [M+H]^+^ | 620.3012 | C_37_H_40_N_4_O_5_ | 2.1 | Ethyl phaeophorbide A |
| **68** | 35.05 | 427.3938  [M+H]^+^ | 426.3865 | C_30_H_50_O | 0.8 | *β*-Amyrin |

–A– represents an A-type interflavan linkage.

Supplementary Table 5. Proposed compounds for seasonal harvests of *S. marginata* extracts of by UHPLC-(ESI)-HRMS.

| **No.** | **Rt (min)** | **Adduct (*m/z*)** | **MW** | **Chemical formula** | **Error (ppm)** | **Annotation of metabolite class or compound** |
| --- | --- | --- | --- | --- | --- | --- |
| **1** | 1.19 | 153.0186  [M-H]^-^ | 154.0259 | C_7_H_6_O_4_ | -4.6 | Protocatecuic acid |
| **2** | 1.19 | 179.0557  [M-H]^-^ | 180.0629 | C_6_H_12_O_6_ | -2.5 | Sugar |
| **3** | 1.44 | 191.0555  [M-H]^-^ | 192.0627 | C_7_H_12_O_6_ | -1.2 | Quinic acid |
| **4** | 1.99 | 315.0724  [M-H]^-^ | 316.0797 | C_13_H_16_O_9_ | 0.9 | *O*-Protocatechuoyl-hexose |
| **5** | 4.20 | 453.1386  [M+H]^+^ | 452.1314 | C_21_H_24_O_11_ | -1.1 | (epi)catechin-*O*-hexose |
| **6** | 4.26 | 355.1018  [M+H]^+^ | 354.0945 | C_16_H_18_O_9_ | -1.6 | *O*-Caffeoylquinic acid |
| **7** | 4.99 | 579.1500  [M+H]^+^ | 578.1428 | C_30_H_26_O_12_ | 0.6 | (epi)catechin-(epi)catechin |
| **8** | 5.70 | 291.0860  [M+H]^+^ | 290.0786 | C_15_H_14_O_6_ | -1.3 | (epi)catechin |
| **9** | 5.81 | 339.1072  [M+H]^+^ | 338.0999 | C_16_H_18_O_8_ | -0.7 | *O*-Coumaroylquinic acid |
| **10** | 5.94 | 865.1970  [M+H]^+^ | 864.1897 | C_45_H_36_O_18_ | -0.6 | (epi)catechin-A-(epi)catechin-(epi)catechin |
| **11** | 6.12 | 563.1543  [M+H]^+^ | 562.1470 | C_30_H_26_O_11_ | -0.9 | (epi)afzelechin-(epi)catechin |
| **12** | 6.12 | 1155.2719  [M+H]^+^ | 1154.2641 | C_60_H_50_O_24_ | -4.4 | (epi)catechin-(epi)catechin-(epi)catechin-(epi)catechin |
| **13** | 6.13 | 1153.2602  [M+H]^+^ | 1152.2530 | C_60_H_48_O_24_ | -0.5 | (epi)catechin-A-(epi)catechin-(epi)catechin-(epi)catechin |
| **14** | 6.15 | 179.0345  [M-H]^-^ | 180.0417 | C_9_H_8_O_4_ | -2.9 | Caffeic acid |
| **15** | 6.39 | 355.1020  [M+H]^+^ | 354.0948 | C_16_H_18_O_9_ | -1.0 | *O*-Caffeoylquinic acid |
| **16** | 6.40 | 565.1550  [M+H]^+^ | 564.1477 | C_26_H_28_O_14_ | -0.3 | Apigenin-*C*-hexose-*O*-pentose |
| **17** | 6.94 | 369.1178  [M+H]^+^ | 368.1105 | C_17_H_20_O_9_ | -0.6 | *O*-Feruloylquinic acid |
| **18** | 7.01 | 1153.2601  [M+H]^+^ | 1152.2528 | C_60_H_48_O_24_ | -0.6 | (epi)catechin-A-(epi)catechin-(epi)catechin-(epi)catechin |
| **19** | 7.09 | 1441.3228  [M+H]^+^ | 1440.3155 | C_75_H_60_O_30_ | -1.0 | (epi)catechin-A-(epi)catechin-(epi)catechin-(epi)catechin-(epi)catechin |
| **20** | 7.27 | 339.1073  [M+H]^+^ | 338.1000 | C_16_H_18_O_8_ | -0.4 | *O*-Coumaroylquinic acid |
| **21** | 7.33 | 597.1451  [M+H]^+^ | 596.1378 | C_26_H_28_O_16_ | 0.2 | Quercetin-*O*-hexose-*O*-pentose |
| **22** | 7.44 | 551.1394  [M+H]^+^ | 550.1321 | C_25_H_26_O_14_ | -0.3 | Luteolin-*C*-pentose-*O*-pentose |

Supplementary Table 5. *(continued)*

| **No.** | **Rt (min)** | **Adduct (*m/z*)** | **MW** | **Chemical formula** | **Error (ppm)** | **Annotation of metabolite class or compound** |
| --- | --- | --- | --- | --- | --- | --- |
| **23** | 7.47 | 419.0973  [M+H]^+^ | 418.0901 | C_20_H_18_O_10_ | 0.1 | Luteolin-8-*C*-pentose |
| **24** | 7.50 | 433.1129  [M+H]^+^ | 432.1056 | C_21_H_20_O_10_ | -0.1 | Kaempferol-*O*-deoxyhexose |
| **25** | 7.56 | 609.1479  [M-H]^-^ | 610.1552 | C_27_H_30_O_16_ | 2.9 | Quercetin-*O*-hexose-*O*-deoxyhexose |
| **26** | 7.61 | 867.2117  [M+H]^+^ | 866.2045 | C_45_H_38_O_18_ | -1.6 | (epi)catechin-(epi)catechin-(epi)catechin |
| **27** | 7.77 | 1153.2598  [M+H]^+^ | 1152.2525 | C_60_H_48_O_24_ | -0.9 | (epi)catechin-A-(epi)catechin-(epi)catechin-(epi)catechin |
| **28** | 7.84 | 577.1343  [M+H]^+^ | 576.1264 | C_30_H_24_O_12_ | 0.4 | (epi)catechin-A-(epi)catechin |
| **29** | 7.85 | 465.1027  [M+H]^+^ | 464.0954 | C_21_H_20_O_12_ | -0.1 | Quercetin-*O*-hexose |
| **30** | 7.91 | 577.1342 [M+H]^+^ | 576.1270 | C_30_H_24_O_12_ | 0.4 | (epi)catechin-A-(epi)catechin |
| **31** | 8.31 | 403.1023  [M+H]^+^ | 402.0950 | C_20_H_18_O_9_ | -0.2 | Apigenin-*C*-pentose |
| **32** | 8.48 | 1103.5310  [M-H]^-^ | 1104.5383 | C_53_H_84_O_24_ | 2.8 | 3-*O*-pentose-23-oxo-olean-12-en-28-oic acid acid, 28-*O-*(hexose-hexose-hexose) ester |
| **33** | 8.55 | 419.0971  [M+H]^+^ | 418.0898 | C_20_H_18_O_10_ | -0.5 | Luteolin-6-*C*-pentose |
| **34** | 8.65 | 863.1810  [M+H]^+^ | 862.1738 | C_45_H_34_O_18_ | -0.9 | (epi)catechin-A-(epi)catechin-A-(epi)catechin |
| **35** | 8.67 | 563.1761  [M+H]^+^ | 562.1689 | C_27_H_30_O_13_ | 0.4 | Apigenin-*C*-boivinopiranose-*O*-hexose |
| **36** | 8.85 | 595.1658  [M+H]^+^ | 594.1586 | C_27_H_30_O_15_ | 0.1 | Kaempferol-*O*-hexose-*O*-deoxyhexose |
| **37** | 9.00 | 577.1551  [M+H]^+^ | 576.1478 | C_27_H_28_O_14_ | -0.2 | Cassiaoccidentalin B |
| **38** | 9.40 | 505.1341  [M+H]^+^ | 504.1268 | C_24_H_24_O_12_ | 0.02 | Di-*O*-caffeoyl hexose |
| **39** | 9.49 | 275.0912  [M+H]^+^ | 274.0839 | C_15_H_14_O_5_ | -0.7 | (epi)afzelechin |
| **40** | 9.57 | 369.1178  [M+H]^+^ | 368.1105 | C_17_H_20_O_9_ | -0.6 | *O*-Feruloylquinic acid |
| **41** | 9.78 | 339.1072  [M+H]^+^ | 338.0999 | C_16_H_18_O_8_ | -0.6 | *O*-Coumaroylquinic acid |
| **42** | 9.85 | 579.1707  [M+H]^+^ | 578.1634 | C_27_H_30_O_14_ | -0.2 | Apigenin-*C*-hexose-*O*-deoxyhexose |
| **43** | 9.89 | 515.1208  [M-H]^-^ | 516.1280 | C_25_H_24_O_12_ | 2.5 | Di-*O*-caffeoylquinic acid |
| **44** | 9.89 | 561.1603  [M+H]^+^ | 560.1530 | C_27_H_28_O_13_ | 0.01 | Tetrastigma B |
| **45** | 9.91 | 863.1806  [M+H]^+^ | 862.1733 | C_45_H_34_O_18_ | -1.4 | (epi)catechin-A-(epi)catechin-A-(epi)catechin |

Supplementary Table 5. *(continued)*

| **No.** | **Rt (min)** | **Adduct (*m/z*)** | **MW** | **Chemical formula** | **Error (ppm)** | **Annotation of metabolite class or compound** |
| --- | --- | --- | --- | --- | --- | --- |
| **46** | 10.04 | 457.1130  [M+H]^+^ | 456.1058 | C_23_H_20_O_10_ | 0.2 | (epi)catechin methylgallate |
| **47** | 10.35 | 417.1177  [M+H]^+^ | 416.1104 | C_21_H_20_O_9_ | -0.8 | Apigenin-8-*C*-deoxyhexose |
| **48** | 10.37 | 561.1602  [M+H]^+^ | 560.1529 | C_27_H_28_O_13_ | -0.1 | Cassiaoccidentalin A |
| **49** | 10.63 | 515.1208  [M-H]^-^ | 516.1281 | C_25_H_24_O_12_ | 2.6 | Di-*O*-caffeoylquinic acid |
| **50** | 10.96 | 487.1235  [M+H]^+^ | 486.1162 | C_24_H_22_O_11_ | -0.01 | Dimethyl (epi)gallocatechin gallate |
| **51** | 11.12 | 417.1177  [M+H]^+^ | 416.1104 | C_21_H_20_O_9_ | -0.7 | Apigenin-6-*C*-deoxyhexose |
| **52** | 11.38 | 591.1708  [M+H]^+^ | 590.1635 | C_28_H_30_O_14_ | -0.1 | Cassiaoccidentalin C |
| **53** | 12.14 | 343.0810  [M+H]^+^ | 342.0738 | C_18_H_14_O_7_ | -0.6 | Dicaffeic acid |
| **54** | 12.31 | 343.0810  [M+H]^+^ | 342.0737 | C_18_H_14_O_7_ | -0.7 | Dicaffeic acid |
| **55** | 12.89 | 457.1126  [M+H]^+^ | 456.1054 | C_23_H_20_O_10_ | -0.6 | (epi)catechin methylgallate |
| **56** | 14.16 | 443.0970  [M+H]^+^ | 442.0897 | C_22_H_18_O_10_ | -0.7 | (epi)catechin gallate |
| **57** | 14.20 | 315.0860  [M+H]^+^ | 314.0788 | C_17_H_14_O_6_ | -0.9 | Dihydroxy-dimethoxyflavone |
| **58** | 15.34 | 443.0969  [M+H]^+^ | 442.0896 | C_22_H_18_O_10_ | -0.8 | (epi)catechin gallate |
| **59** | 15.51 | 1043.5452  [M-H]^-^ | 1044.5525 | C_52_H_84_O_21_ | 1.9 | 3-*O*-(pentose-hexose-deoxyhexose-pentose) hederagenin |
| **60** | 15.53 | 941.5128  [M-H]^-^ | 942.5201 | C_48_H_78_O_18_ | 1.4 | 3-*O*-(hexose-hexose-deoxyhexose) hederagenin |
| **61** | 15.72 | 1057.5609  [M-H]^-^ | 1058.5681 | C_53_H_86_O_21_ | 1.9 | 3-*O*-(deoxyhexose-pentose) hederagenin-28-*O-*(deoxyhexose-hexose) ester |
| **62** | 15.91 | 911.5031  [M-H]^-^ | 912.5104 | C_47_H_76_O_17_ | 2.3 | 3-*O*-(pentose-deoxyhexose-hexose) hederagenin |
| **63** | 15.99 | 499.3436  [M-H]^-^ | 500.3509 | C_31_H_48_O_5_ | 1.5 | olean-12-ene-23,28-dioic acid, 30-methyl ester |
| **64** | 16.26 | 1041.5298  [M-H]^-^ | 1042.5370 | C_52_H_82_O_21_ | 2.1 | 3-*O*-(deoxyhexose-pentose)-30-norolean-12,20(29)-dien-28-*O*-(hexose-hexose) ester |
| **65** | 16.61 | 1055.5454  [M-H]^-^ | 1056.5527 | C_53_H_84_O_21_ | 2.1 | 3-*O*-(hexose-pentose-deoxyhexose-pentose)-23-oxo-olean-12-en-28-oic acid |
| **66** | 16.82 | 909.4877  [M-H]^-^ | 910.4950 | C_47_H_74_O_17_ | 2.6 | 3-*O*-(pentose-hexose)-23-oxo-olean-12-en-28-oic acid, 28-*O-*(deoxyhexose) ester |
| **67** | 17.42 | 1027.5503  [M-H]^-^ | 1028.5576 | C_52_H_84_O_20_ | 2.0 | 3-*O*-(pentose-deoxyhexose-hexose-pentose) oleanolic acid |

Supplementary Table 5. *(continued)*

| **No.** | **Rt (min)** | **Adduct (*m/z*)** | **MW** | **Chemical formula** | **Error (ppm)** | **Annotation of metabolite class or compound** |
| --- | --- | --- | --- | --- | --- | --- |
| **68** | 17.54 | 1057.5595  [M-H]^-^ | 1058.5668 | C_53_H_86_O_21_ | 0.6 | 3-*O*-pentose oleanolic acid-28-*O-*(hexose-hexose-deoxyhexose) ester |
| **69** | 17.62 | 1029.5660  [M-H]^-^ | 1030.5737 | C_52_H_86_O_20_ | 2.5 | stigmasta-5,22-dien-3-ol 3-*O*-(hexose-hexose-hexose-pentose) |
| **70** | 17.66 | 895.5082  [M-H]^-^ | 896.5155 | C_47_H_76_O_16_ | 2.4 | 3-*O*-(pentose-deoxyhexose-hexose) oleanolic acid |
| **71** | 17.69 | 1027.5502  [M-H]^-^ | 1028.5575 | C_52_H_84_O_20_ | 1.9 | 3-*O*-(pentose-deoxyhexose-pentose-deoxyhexose) hederagenin |
| **72** | 17.69 | 351.1071  [M+H]^+^ | 350.0997 | C_17_H_18_O_8_ | -1.1 | Caffeoylquinic acid derivative |
| **73** | 17.70 | 199.0600  [M-H]^-^ | 198.0527 | C_9_H_10_O_5_ | -0.7 | Syringic acid |
| **74** | 17.70 | 357.1175  [M+H]^+^ | 356.1102 | C_16_H_20_O_9_ | -1.4 | *O*-Feruloyl hexose |
| **75** | 17.71 | 897.5238  [M-H]^-^ | 898.5310 | C_47_H_78_O_16_ | 2.3 | stigmasta-5,22-dien-3-ol 3-*O*-(hexose-hexose-hexose) |
| **76** | 17.92 | 369.1177  [M+H]^+^ | 368.1104 | C_17_H_20_O_9_ | -0.9 | *O*-Feruloylquinic acid |
| **77** | 18.07 | 1041.5654  [M-H]^-^ | 1042.5727 | C_53_H_86_O_20_ | 1.4 | 3-*O*-(deoxyhexose-deoxyhexose-hexose oleanolic acid |
| **78** | 18.41 | 895.5082  [M-H]^-^ | 896.5154 | C_47_H_76_O_16_ | 2.3 | 3-*O*-(deoxyhexose-deoxyhexose-pentose) hederagenin |
| **79** | 19.75 | 865.4971  [M-H]^-^ | 866.5044 | C_46_H_74_O_15_ | 1.9 | 3-*O*-(pentose-deoxyhexose-pentose) oleanolic acid |
| **80** | 20.84 | 733.4550  [M-H]^-^ | 734.4623 | C_41_H_66_O_11_ | 2.4 | 3-*O*-(pentose-deoxyhexose) oleanolic acid |
| **81** | 21.02 | 1011.5554  [M-H]^-^ | 1012.5627 | C_52_H_84_O_19_ | 2.0 | 3-*O*-(pentose-deoxyhexose-deoxyhexose-pentose) oleanolic acid |

–A– represents an A-type interflavan linkage.

Supplementary Table 6. Discriminant metabolites that contribute to the differentiation of the seasonal harvests for the MS-NMR fused data features of *B. intermedia* samples.

| **ID** | ***δ* (ppm)** | **Multiplicity, *J* in Hz** | **Type of hydrogen** | **Annotation of metabolite class or compound** | ***p*-value** | **FDR^a^** |
| --- | --- | --- | --- | --- | --- | --- |
| **137** | 7.53 | d, *J* = 2.3 | Aromatic, Ar-H | Quercetin derivatives | 1.92E-12 | 1.28E-13 |
| **153** | 6.90 | s | Aromatic, Ar-H | Galloyl group | 7.88E-05 | 1.05E-05 |
| **292** | 1.35 | m | Alkyl (methylene), -CH_2_ | Triterpene | 1.04E-04 | 2.08E-05 |
| **266** | 2.39 | s | *α* to oxygen, -CH-OH | Quinic acid derivatives | 2.72E-03 | 7.25E-04 |
| **150** | 7.04 | s | Aromatic, Ar-H | Galloyl group | 4.91E-03 | 1.64E-03 |
| **248** | 3.12 | dd, *J* = 9.7, 2.8 | Alkyl (methylene), -CH_2_ | Sacharide unit | 5.29E-03 | 2.12E-03 |
| **241** | 3.37 | t, *J* = 9.6 | *α* to oxygen, -CH-OH | Sacharide unit | 1.30E-02 | 6.07E-03 |
| **170** | 6.21 | d, *J* = 2.1 | Aromatic, Ar-H | Quercetin derivatives | 2.12E-02 | 1.13E-02 |
| **235** | 3.62 | m | *α* to oxygen, -CH-OH | Sacharide unit | 2.12E-02 | 1.27E-02 |
| **307** | 0.77 | s | Alkyl (methyl), -CH_3_ | Triterpene | 5.08E-02 | 3.39E-02 |
| **295** | 1.24 | s | Alkyl (methyl), -CH_3_ | Triterpene | 7.60E-02 | 5.57E-02 |
| **300** | 1.04 | d, *J* = 6.1 | Alkyl (methyl), -CH_3_ | Sacharide unit (deoxysugar) | 1.24E-01 | 9.92E-02 |
| **301** | 1.00 | s | Alkyl (methyl), -CH_3_ | Triterpene | 8.53E-02 | 7.39E-02 |
| **152** | 6.93 | s | Aromatic, Ar-H | Galloyl group | 1.17E-01 | 1.09E-01 |
| **155** | 6.83 | d, *J* = 8.5 | Aromatic, Ar-H | Quercetin derivatives | 2.40E-01 | 2.40E-01 |

**^a^** FDR: False Discovery Rate.

s= singlet; d= doublet; dd= doublet of doublet; t= triplet; q=quartet; m= multiplet; br s = broad singlet

The splitting pattern has been suggested based on the result of *J*-resolved.

Supplementary Table 7. Discriminant metabolites that contribute to the differentiation of the seasonal harvests for the MS-NMR fused data features of *S. marginata* samples.

| **ID** | ***δ* (ppm)** | **Multiplicity, *J* in Hz** | **Type of hydrogen** | **Annotation of metabolite class or compound** | ***p*-value** | **FDR^a^** |
| --- | --- | --- | --- | --- | --- | --- |
| **151** | 3.99 | m | *α* to oxygen, -CH-OH | Sacharide unit | 4.45E-03 | 2.97E-04 |
| **163** | 3.52 | dd, *J* = 11.2, 3.6 | *α* to oxygen, -CH-OH | Sacharide unit | 4.45E-03 | 5.94E-04 |
| **182** | 2.72 | dd, *J* = 13.0, 3.6 | Alkyl (methylene), -CH_2_ | Sapogenin | 4.45E-03 | 8.90E-04 |
| **194** | 2.27 | m | Alkyl (methylene), -CH_2_ | Sapogenin | 4.45E-03 | 1.19E-03 |
| **213** | 1.49 | s | Alkyl (methyl), -CH_3_ | Sapogenin | 4.45E-03 | 1.48E-03 |
| **121** | 5.19 | d, *J* = 3.7 | *α*-anomeric proton, -CH-OH | Sacharide unit | 4.60E-03 | 1.84E-03 |
| **175** | 3.03 | t, *J* = 9.0 | *α* to oxygen, -CH-OH | Sacharide unit | 6.38E-03 | 2.98E-03 |
| **224** | 1.08 | d, *J* = 6.1 | Alkyl (methyl), -CH_3_ | Sacharide unit (deoxysugar) | 3.24E-02 | 1.73E-02 |
| **161** | 3.58 | m | *α* to oxygen, -CH-OH | Sacharide unit | 3.27E-02 | 1.96E-02 |
| **171** | 3.18 | br s | Alkyl (methylene), -CH | Sapogenin | 5.82E-02 | 3.88E-02 |
| **191** | 2.39 | m | Alkyl (methylene), -CH | Sapogenin | 6.85E-02 | 5.03E-02 |
| **178** | 2.91 | m | Alkyl (methylene), -CH_2_ | Sapogenin | 8.83E-02 | 7.06E-02 |
| **144** | 4.26 | d, *J* = 7.9 | *β*-anomeric proton, -CH-OH | Sacharide unit | 9.92E-02 | 8.60E-02 |
| **103** | 5.90 | d, *J* = 2.2 | Aromatic, Ar-H | Catechin derivatives | 1.01E-01 | 9.45E-02 |
| **205** | 1.82 | m | Alkyl (methylene), -CH_2_ | Sapogenin | 1.07E-01 | 1.07E-01 |

**^a^** FDR: False Discovery Rate.

s= singlet; d= doublet; dd= doublet of doublet; t= triplet; q=quartet; m= multiplet; br s = broad singlet

The splitting pattern has been suggested based on the result of *J*-resolved.

Supplementary Table 8. Discriminant metabolites that contribute to the differentiation of the seasonal harvests for the HRMS data features of *B. intermedia* samples.

| **MZmine**  **ID^a^** | **Rt**  **(min)** | **Adduct (*m/z*)** | **Chemical**  **Formula (RDB)** | **Annotation of metabolite class or compound^b^** | ***p*-value** | **FDR^c^** |
| --- | --- | --- | --- | --- | --- | --- |
| **N2** | 26.88 | 455.3546 [M-H]^-^ | C_30_H_48_O_3_ (RDB = 7) | Betulinic acid | 8.72E-04 | 5.81E-05 |
| **N23** | 27.61 | 455.3547 [M-H]^-^ | C_30_H_48_O_3_ (RDB = 7) | Oleanolic acid | 1.85E-03 | 2.47E-04 |
| **P4128** | 35.05 | 427.3938 [M+H]^+^ | C_30_H_50_O (RDB = 6) | *β*-amyrin | 2.72E-03 | 5.44E-04 |
| **P22** | 27.65 | 439.3576 [M+H]^+^ | C_30_H_46_O_2_ (RDB = 8) | 3-oxo-olean-12-en-28-al | 8.57E-03 | 2.29E-03 |
| **P15** | 6.49 | 303.0865 [M+H]^+^ | C_16_H_14_O_6_ (RDB = 10) | Flavonoid | 9.45E-03 | 3.15E-03 |
| **P13** | 7.78 | 465.1035 [M+H]^+^ | C_21_H_20_O_12_ (RDB = 12) | Quercetin-*O*-hexose | 5.53E-02 | 2.21E-02 |
| **N113** | 5.40 | 647.0914 [M-H]^-^ | [C_28_H_24_O_18_](https://pubchem.ncbi.nlm.nih.gov/#query=C28H24O18) (RDB = 17) | Tri-*O*-galloylquinic acid | 6.08E-02 | 2.84E-02 |
| **P76** | 5.41 | 497.0932 [M+H]^+^ | C_21_H_20_O_14_ (RDB = 12) | Di-*O*-galloylquinic acid | 7.01E-02 | 3.74E-02 |
| **N25** | 7.97 | 615.1018 [M-H]^-^ | C_28_H_24_O_16_ (RDB = 17) | Quercetin-*O*-(*O*-galloyl)-hexose | 7.50E-02 | 4.50E-02 |
| **P30** | 7.97 | 617.1147 [M+H]^+^ | C_28_H_24_O_16_ (RDB = 17) | Quercetin-*O*-(*O*-galloyl)-hexose | 1.58E-01 | 1.05E-01 |
| **N1103** | 5.85 | 799.1035 [M-H]^-^ | C_33_H_41_O_20_ (RDB = 22) | Tetra-*O*-galloylquinic acid | 1.76E-01 | 1.29E-01 |
| **P11** | 8.40 | 435.0928 [M+H]^+^ | C_20_H_18_O_11_ (RDB = 12) | Quercetin-*O*-pentose | 2.14E-01 | 1.71E-01 |
| **P153** | 5.40 | 479.0826 [M+H]^+^ | C_21_H_18_O_13_ (RDB = 13) | Di-*O*-galloylshikimic acid | 5.80E-01 | 5.03E-01 |
| **N170** | 5.41 | 495.0797 [M-H]^-^ | C_21_H_20_O_14_ (RDB = 12) | Di-*O*-galloylquinic acid | 5.87E-01 | 5.48E-01 |
| **N181** | 5.74 | 799.1032 [M-H]^-^ | C_33_H_41_O_20_ (RDB = 22) | Tetra-*O*-galloylquinic acid | 6.02E-01 | 6.02E-01 |

**^a^** MZMine ID includes: N = negative ionization polarity and P = positve ionization polarity.

**^b^** RDB = Ring Double Bond equivalent.

**^c^** FDR: False Discovery Rate.

Supplementary Table 9. Discriminant metabolites that contribute to the differentiation of the seasonal harvests for the HRMS data features of *S. marginata* samples.

| **MZmine**  **ID^a^** | **Rt**  **(min)** | **Adduct (*m/z*)** | **Chemical**  **Formula (RDB)** | **Annotation of metabolite class or compound^b^** | ***p*-value** | **FDR^c^** |
| --- | --- | --- | --- | --- | --- | --- |
| **N2053** | 17.71 | 897.5238 [M-H]^-^ | C_47_H_78_O_16_ (RDB = 9) | stigmasta-5,22-dien-3-ol 3-*O*-(hexose-hexose-hexose) | 1.97E-03 | 1.32E-04 |
| **N1452** | 17.62 | 1029.5660 [M-H]^-^ | C_52_H_86_O_20_ (RDB = 10) | stigmasta-5,22-dien-3-ol 3-*O*-(hexose-hexose-hexose-pentose) | 1.86E-02 | 2.48E-03 |
| **N237** | 15.72 | 1057.5609 [M-H]^-^ | C_53_H_86_O_21_ (RDB = 9) | 3-*O*-(deoxyhexose-pentose) hederagenin-28-*O-*(deoxyhexose-hexose) ester | 6.41E-02 | 1.28E-02 |
| **N1183** | 18.41 | 896.0099 [M-H]^-^ | C_25_H_19_O_30_N_7_ (RDB = 20) | *N*-containing compound | 7.24E-02 | 1.93E-02 |
| **P1907** | 10.46 | 561.1602 [M+H]^+^ | C_27_H_28_O_13_ (RDB = 14) | Cassiaoccidentalin A | 7.95E-02 | 2.65E-02 |
| **N2411^d^** | 18.40 | 896.5109 [M-H+1]^-^ | C_47_H_76_O_16_ (RDB = 10) | 3-*O*-(deoxyhexose-deoxyhexose-pentose) hederagenin | 8.03E-02 | 3.21E-02 |
| **N43** | 18.41 | 895.5082 [M-H]^-^ | C_47_H_76_O_16_ (RDB = 10) | 3-*O*-(deoxyhexose-deoxyhexose-pentose) hederagenin | 8.20E-02 | 3.83E-02 |
| **P372** | 18.44 | 439.3568 [M+H]^+^ | C_30_H_46_O_2_ (RDB = 8) | Triterpene | 8.66E-02 | 4.62E-02 |
| **N1182** | 18.41 | 1793.0229 [2M-H]^-^ | - | Complex of *m/z* 895.5082 and *m/z* 896.5109 | 8.98E-02 | 5.39E-02 |
| **N40** | 15.91 | 911.5031 [M-H]^-^ | C_47_H_76_O_17_ (RDB = 10) | 3-*O*-(pentose-deoxyhexose-hexose) hederagenin | 1.81E-01 | 1.20E-01 |
| **N1181^d^** | 17.69 | 1029.5565 [M-H+2]^-^ | C_52_H_84_O_20_ (RDB = 11) | 3-*O*-(pentose-deoxyhexose-pentose-deoxyhexose) hederagenin | 2.58E-01 | 1.89E-01 |
| **N2114** | 17.69 | 1027.5502 [M-H]^-^ | C_52_H_84_O_20_ (RDB = 11) | 3-*O*-(pentose-deoxyhexose-pentose-deoxyhexose) hederagenin | 2.61E-01 | 2.08E-01 |
| **N41^d^** | 17.69 | 1028.5536 [M-H+1]^-^ | C_52_H_84_O_20_ (RDB = 11) | 3-*O*-(pentose-deoxyhexose-pentose-deoxyhexose) hederagenin | 2.95E-01 | 2.56E-01 |
| **P1906** | 9.89 | 561.1603 [M+H]^+^ | C_27_H_28_O_13_ (RDB = 14) | Tetrastigma B | 3.87E-01 | 3.61E-01 |
| **N42** | 15.51 | 1043.5452 [M-H]^-^ | C_52_H_84_O_21_ (RDB = 11) | 3-*O*-(pentose-hexose-deoxyhexose-pentose) hederagenin | 8.84E-01 | 8.84E-01 |

**^a^** MZMine ID includes: N = negative ionization polarity and P = positve ionization polarity.

**^b^** RDB = Ring Double Bond equivalent.

**^c^** FDR: False Discovery Rate.

**^d^** Isotopologues of ion adducts with one [M-H+1]^-^ or two [M-H+2]^-^ isotopes in the molecule (^13^C, ^2^H or ^17^O).
